# Baseline severity and the prediction of placebo response in clinical trials for alcohol dependence: A meta‐regression analysis to develop an enrichment strategy

**DOI:** 10.1111/acer.14670

**Published:** 2021-08-21

**Authors:** Bruno Scherrer, Julien Guiraud, Giovanni Addolorato, Henri‐Jean Aubin, Andrea de Bejczy, Amine Benyamina, Wim van den Brink, Fabio Caputo, Maurice Dematteis, Anna E. Goudriaan, Antoni Gual, Falk Kiefer, Lorenzo Leggio, Otto‐Michael Lesch, Icro Maremmani, David J. Nutt, François Paille, Pascal Perney, Roch Poulnais, Quentin Raffaillac, Jürgen Rehm, Benjamin Rolland, Nicolas Simon, Bo Söderpalm, Wolfgang H. Sommer, Henriette Walter, Rainer Spanagel

**Affiliations:** ^1^ Bruno Scherrer Conseil Saint Arnoult en Yvelines France; ^2^ Department of Psychiatry Amsterdam Neuroscience Amsterdam UMC University of Amsterdam Amsterdam The Netherlands; ^3^ DA Pharma Paris France; ^4^ Alcohol Use Disorder and Alcohol Related Disease Unit Department of Internal Medicine and Gastroenterology Fondazione Policlinico Universitario A.Gemelli IRCCS Rome Italy; ^5^ Internal Medicine Unit Department of Internal Medicine and Gastroenterology Columbus‐Gemelli Hospital Fondazione Policlinico Universitario A. Gemelli IRCCS Rome Italy; ^6^ Centre de Recherche en Epidémiologie et Santé des Populations (CESP) French Institute of Health and Medical Research (Inserm) Paris France; ^7^ Addiction Research and Treatment Center Paul Brousse Hospital Paris‐Sud University Villejuif France; ^8^ Section of Psychiatry and Neurochemistry Institute of Neuroscience and Physiology Sahlgrenska Academy University of Gothenburg Gothenburg Sweden; ^9^ Department of Translational Medicine University of Ferrara Ferrara Italy; ^10^ Department of Translational Medicine Center for the Study and Treatment of Alcohol‐Related Diseases University of Ferrara Ferrara Italy; ^11^ Department of Translational Medicine Center for the Study and Treatment of Chronic Inflammatory Bowel Diseases (IBD) and Gastroenterological Manifestations of Rare Diseases University of Ferrara Ferrara Italy; ^12^ Department of Internal Medicine Santissima Annunziata Hospital Cento (Ferrara) University of Ferrara Ferrara Italy; ^13^ Department of Addiction Medicine Faculty of Medicine Grenoble Alpes University Hospital Grenoble Alpes University Grenoble France; ^14^ Department of Research and Quality of Care Arkin Amsterdam The Netherlands; ^15^ Amsterdam Public Health Research Institute Amsterdam The Netherlands; ^16^ Psychiatry Department Neurosciences Institute, Hospital Clinic IDIBAPS Barcelona Spain; ^17^ Medical Faculty Mannheim Central Institute of Mental Health Institute of Psychopharmacology University of Heidelberg Heidelberg Germany; ^18^ Bethania Hospital for Psychiatry, Psychosomatics, and Psychotherapy Greifswald Germany; ^19^ Clinical Psychoneuroendocrinology and Neuropsychopharmacology Section Translational Addiction Medicine Branch National Institute on Drug Abuse Intramural Research Program and National Institute on Alcohol Abuse and Alcoholism Division of Intramural Clinical and Basic Research National Institutes of Health Baltimore and Bethesda Maryland USA; ^20^ Medication Development Program National Institute on Drug Abuse Intramural Research Program National Institutes of Health Baltimore Maryland USA; ^21^ Department of Behavioral and Social Sciences Center for Alcohol and Addiction Studies Brown University Providence Rhode Island USA; ^22^ Division of Addiction Medicine Department of Medicine Johns Hopkins University Baltimore Maryland USA; ^23^ Department of Neuroscience Georgetown Medical Center Washington District of Columbia USA; ^24^ Department of Social Psychiatry University for Psychiatry and Psychotherapy Vienna Austria; ^25^ Santa Chiara University Hospital University of Pisa Pisa Italy; ^26^ Centre for Neuropsychopharmacology Imperial College London London UK; ^27^ Department of Addiction Treatment University Hospital Vandoeuvre‐lès‐Nancy France; ^28^ Addiction Medicine Hospital Grau‐du‐Roi Nimes France; ^29^ Centre for Addiction and Mental Health Institute for Mental Health Policy Research Toronto Ontario Canada; ^30^ Department of Psychiatry Dalla Lana School of Public Health University of Toronto Toronto Ontario Canada; ^31^ Clinical Psychology and Psychotherapy Technical University Dresden Dresden Germany; ^32^ Department of International Health Projects Institute for Leadership and Health Management I.M. Sechenov First Moscow State Medical University Moscow Russia; ^33^ UCBL INSERM U1028 CNRS UMR5292 Centre de Recherche en Neuroscience de Lyon (CRNL) Univ Lyon Bron France; ^34^ Department of Clinical Pharmacology CAP‐TV Aix Marseille Univ APHM INSERM IRD SESSTIM Hop Sainte Marguerite Marseille France; ^35^ Central Institute of Mental Health Institute of Psychopharmacology Heidelberg University Mannheim Germany

**Keywords:** alcohol dependence, alcohol use disorder, placebo response, predictor, randomized controlled trials

## Abstract

**Background:**

There is considerable unexplained variability in alcohol abstinence rates (AR) in the placebo groups of randomized controlled trials (RCTs) for alcohol dependence (AD). This is of particular interest because placebo responses correlate negatively with treatment effect size. Recent evidence suggests that the placebo response is lower in very heavy drinkers who show no “spontaneous improvement” prior to treatment initiation (high‐severity population) than in a mild‐severity population and in studies with longer treatment duration. We systematically investigated the relationship between population severity, treatment duration, and the placebo response in AR to inform a strategy aimed at reducing the placebo response and thereby increasing assay sensitivity in RCTs for AD.

**Methods:**

We conducted a systematic literature review on placebo‐controlled RCTs for AD.We assigned retained RCTs to high‐ or mild‐severity groups of studies based on baseline drinking risk levels and abstinence duration before treatment initiation. We tested the effects of population severity and treatment duration on the placebo response in AR using meta‐regression analysis.

**Results:**

Among the 19 retained RCTs (comprising 1996 placebo‐treated patients), 11 trials were high‐severity and 8 were mild‐severity RCTs. The between‐study variability in AR was lower in the high‐severity than in the mild‐severity studies (interquartile range: 7.4% vs. 20.9%). The AR in placebo groups was dependent on population severity (*p* = 0.004) and treatment duration (*p* = 0.017) and was lower in the high‐severity studies (16.8% at 3 months) than the mild‐severity studies (36.7% at 3 months).

**Conclusions:**

Pharmacological RCTs for AD should select high‐severity patients to decrease the magnitude and variability in the placebo effect and and improve the efficiency of drug development efforts for AD.

## Graphical abstract

Using a meta‐regression analysis, we investigated the effect of baseline population severity and treatment duration on the placebo response in alcohol abstinence rate in 19 randomized controlled trials (RCTs) conducted in the treatment of alcohol dependence (AD). The placebo response was significantly lower in the high‐severity group of studies and in studies with longer treatment duration. Future pharmacological RCTs for AD should consider the selection of high‐severity patients to decrease the placebo effect and increase assay sensitivity.

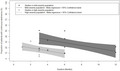

## INTRODUCTION

Alcohol dependence (AD) affects 7.7% and 3.4% of the adult population in the United States of America and in the European Union, respectively (Rehm et al., 2015; World Health Organization, 2018), and accounts for 71% of all alcohol‐related harm and for 60% of all social costs related to alcohol (Rehm et al., 2013). There is strong evidence that alcohol‐related harm is determined by the amount of alcohol consumed and the specific drinking pattern (Rehm et al., 2010). The amount of alcohol consumption has been categorized in different Drinking Risk Levels (DRLs) by the World Health Organization (WHO; World Health Organization, 2000), and subjects with a very high (VH) DRL (see Table S1) are responsible for the majority of AD attributable burden (Hasin et al., 2017; Rehm et al., 2018). Therefore, subjects with a VH DRL constitute a target population of primary concern in the treatment of AD.

One of the AD treatment objectives is the achievement of stable abstinence by prevention of relapse after detoxification (European Medicines Agency, 2010). Approved treatments in the maintenance of alcohol abstinence in the United States of America and in Europe include acamprosate, naltrexone, and disulfiram. In addition, nalmefene has been approved by the European Medicines Agency (EMA) for the reduction of alcohol consumption. However, these proven‐effective medicines only show modest efficacy with many patients not responding to these treatments (European Medicines Agency, 2010; Litten et al., 2012; van den Brink et al., 2018), and thus, there is a need for additional treatments. However, development of medications for the treatment of AD is challenging and the demonstration of efficacy of treatments approved for this indication is based on a mix of positive and negative studies (European Medicines Agency, 2010; Litten et al., 2012; Witkiewitz et al., 2019). One of the main reasons for these mixed results has been the unpredictable variability of the placebo response in randomized controlled trials (RCTs) for AD. In an analysis on 51 naltrexone and acamprosate double‐blind RCTs, the placebo response was significantly negatively correlated with the treatment effect size on total abstinence (Litten et al., 2013). It is recognized that studies often fail when the placebo response is high (European Medicines Agency, 2007). In this context, the development of enrichment strategies for clinical trials for AD will increase the reliability of the expected effect size thanks to decrease of variability of the placebo response and increase of the power of the study thanks to decrease of the placebo effect. It will therefore improve the efficiency of drug development through targeting the treatment to those patients who will benefit the most from pharmacological interventions. Enrichment is the prospective use of any patient characteristic to select a study population in which detection of a drug effect (if in fact present) is more likely than it would be in an unselected population (US Food & Drug Administration, 2019).

Numerous factors potentially predicting placebo response in the treatment of AD have been studied over the last 20 years such as study design and demographic characteristics (Litten et al., 2013). Recent subgroup analyses in studies for the treatment of AD suggest that the placebo response in double‐blind RCTs is higher and treatment effect size is lower in patients with a low or medium DRL at baseline (L/M DRL: see Table S1), in patients with more than 14 consecutive days of abstinence before treatment initiation (“early abstainers”), and/or in patients who reduce their alcohol consumption to a L/M DRL prior to treatment initiation (“early reducers”). Conversely, placebo response is lower and treatment effect size is higher in the complement population which includes AD patients with a high or VH DRL at baseline (H/VH DRL, see Table S1) and who are not early abstainers/reducers (Gual et al., 2013; Gueorguieva et al., 2011, 2012; Mann et al., 2016; van den Brink et al., 2018, 2013, 2014). In other words, and in analyses at patient level, the placebo response seems to be lower and treatment effect size higher in heavy drinkers without spontaneous improvement before treatment initiation. With respect to their level of response to placebo treatment, not early abstainers/reducers with H/VH DRL have been defined in the literature as the high‐severity AD population and L/M DRL or early abstainers/reducers as the mild‐severity AD population (van den Brink et al., 2018). Although the effect of this notion of AD severity has been studied at patient level, it has so far not been systematically investigated at study level. In addition, analyses at patient level showed that the placebo response in RCTs for AD was dependent on treatment duration with higher relapse rates in studies with a longer treatment duration (Anton et al., 1999, 2005; Baltieri & Andrade, 2003; Baltieri et al., 2008; Chick et al., 2000; Kiefer et al., 2003; Pelc et al., 1997; Volpicelli et al., 1997). However, in a previous meta‐analysis of 51 RCTs for AD, the placebo response at study level was not dependent on the unadjusted treatment duration (Litten et al., 2013). Nevertheless, the effect of treatment duration on the placebo response has so far not been adjusted for population severity at study level.

Therefore, the current study systematically and simultaneously investigated the relationships of the placebo response in the maintenance of abstinence in double‐blind RCTs with population severity (high vs. mild severity) and treatment duration to explore whether an enrichment strategy using these potential predictors might help to reduce the variability of the placebo response and increase assay sensitivity in future clinical trials.

## MATERIALS AND METHODS

### Study selection and systematic review

A systematic literature review was performed to select double‐blind placebo‐controlled RCTs investigating the efficacy of approved pharmacological interventions, new chemical entities, or repurposed medications in the maintenance of abstinence in alcohol‐dependent patients and conducted with similar experimental conditions (except for the population severity and the treatment duration).

The Miller et al. (2011) systematic literature review on medical treatment of AD was screened to obtain keywords for pharmacological substances or repurposed medications tested in the treatment of AD. They were reviewed and expanded based on authors’ knowledge and were then used for a systematic literature search by the online portal of the National Library of Medicine (https://pubmed.ncbi.nlm.nih.gov/ including PubMed, PubMed Central, and MEDLINE. A systematic screening of the original articles published until October 1, 2020, was performed based on PRISMA guidelines, and the keywords and combinations are provided in the supplementary methods. In addition, the reference sections of identified papers as well as review and meta‐analysis articles were screened for further relevant citations. Three reviewers (JG, RP, and QR) independently screened titles and abstracts of articles and read the full text of papers deemed potentially eligible by either reviewer. Reviewer disagreements were solved by discussion, and consensus was reached in all instances. Only peer‐reviewed original articles written in English were retained if they fulfilled inclusion/noninclusion criteria.

Only comparative, parallel arms, double‐blind, randomized, placebo‐controlled (oral) medication trials conducted to maintain abstinence from alcohol were eligible. Included studies enrolled alcohol‐dependent patients as diagnosed with DSM (IV or earlier), ICD (10 or earlier), or equivalent criteria. Studies enrolling patients with cooccurring disorders (severe psychiatric comorbidities, polydrug or other substance use disorders (except tobacco), or severe hepatic dysfunction (liver cirrhosis, HCV) were excluded. Patients had to be abstinent before starting the study medication and to be monitored in an outpatient setting during the treatment phase.

Only studies reporting the abstinence rate were included. Abstinence rate is the primary endpoint recommended by the US Food Drug Administration and the EMA for demonstration of efficacy in the maintenance of abstinence (European Medicines Agency, 2010; US Food & Drug Administration, 2015). Our definition of abstinence was continuous abstinence (no relapse to any alcohol use) throughout the treatment period. Studies with other outcome definitions were excluded. Dropouts were treated as treatment failures (patient not continuously abstinent). The following information was extracted in duplicate using a data collection form, which has been piloted, from each retained study: (1) treatment duration, (2) alcohol consumption prior to screening in the placebo arm, (3) abstinence duration before randomization, (4) total number of patients allocated to the placebo arm, (5) total number of continuously abstinent patients in the placebo arm at end of treatment, and (6) any other reported baseline characteristics. Data from the retained studies were then used to assign studies to the group of high‐severity or the group of mild‐severity studies.

### Allocation of studies in each population

Assignment of studies to the group of high‐severity or the group of mild‐severity studies was based on the two criteria defined in the literature for trials directed at maintenance of abstinence: level of alcohol consumption at baseline and abstinence duration before treatment initiation.

**FIGURE 1:**
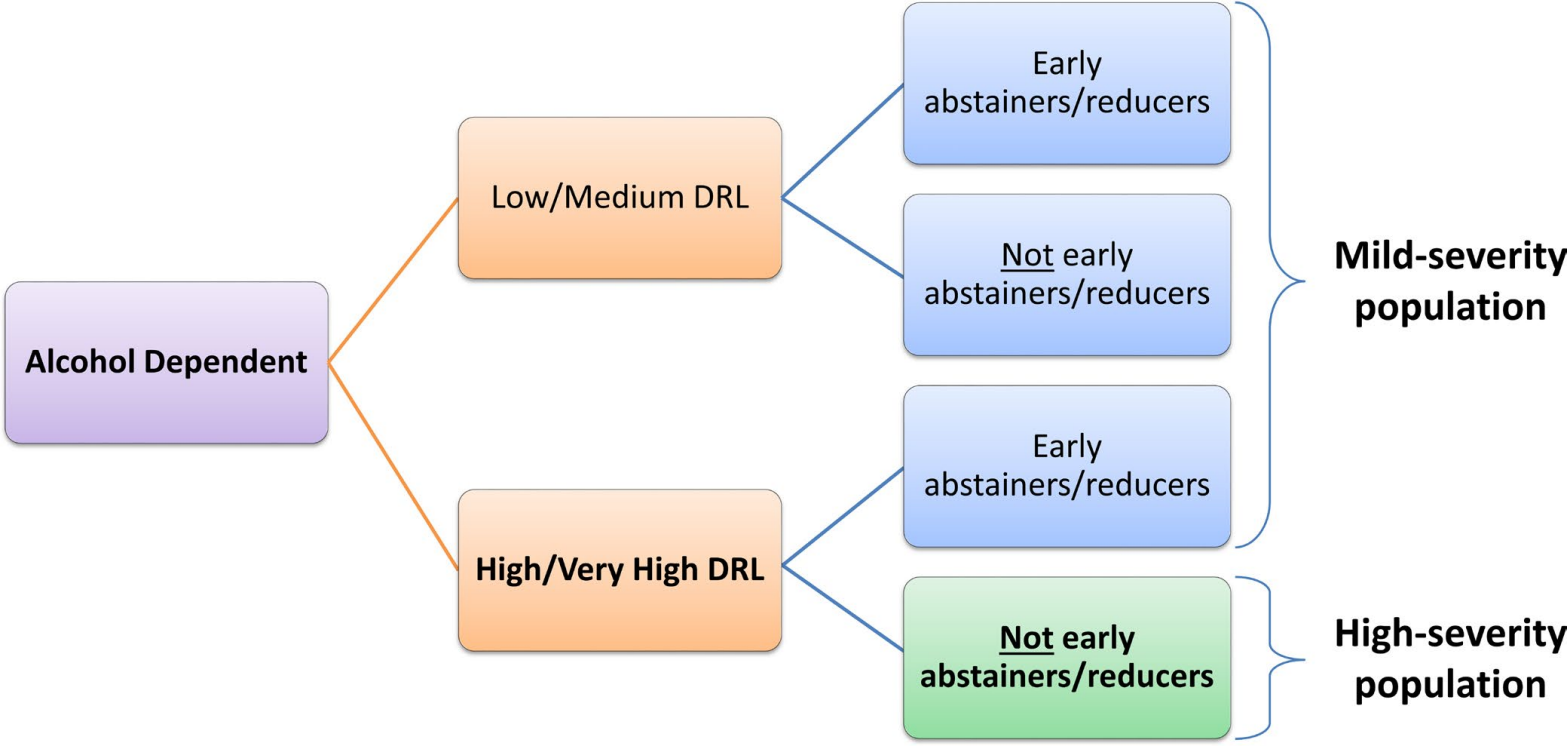
Definitions of alcohol‐dependent subpopulations according to van den Brink et al. (2018)

The first criterion is based on WHO DRL to categorize patients depending on their mean alcohol consumption (in grams of pure alcohol per day) at baseline (Figure 1; Table S1). If the reported mean alcohol consumption at baseline in the placebo group was lower than the medium DRL threshold (60 g alcohol/day for men and 40 g alcohol/day for women), the study was considered as being conducted in the L/M DRL population. If only the mean number of standard drinks at baseline in the placebo group was reported, then the conversion to grams was performed using the following country‐specific standard drinking units: South Korea 8 g; Australia, Belgium, France 10 g; Italy 12 g; United States of America 14 g; and Germany 15 g (World Health Organization, 2018). RCTs conducted in L/M DRL populations were allocated to the mild‐severity population regardless of the abstinence duration prior to treatment (Figure 1).

For RCTs not categorized as L/M DRL studies, a second criterion linked to the abstinence duration before treatment initiation was applied, which allowed to distinguish not early abstainers from other patients (Figure 1). If the inclusion/exclusion criteria specified a detoxification period of less than 14 days prior to treatment initiation, the study was considered to be conducted in not early abstainers. Conversely, studies with inclusion/exclusion criteria specifying a detoxification period longer than 14 days were considered to be conducted in early abstainers. The mean detoxification or pretreatment abstinence duration was used in case it was not possible to classify the study based on the inclusion/exclusion criteria. Studies with a mean detoxification or pretreatment abstinence duration ≤11 days were considered as being conducted in not early abstainers. Conversely, studies with a mean detoxification or pretreatment abstinence duration ≥17 days were considered as conducted in early abstainers. Studies with a mean detoxification or pretreatment abstinence period between 11 and 17 days were excluded as it was too close from the 14 days threshold, and thus, we considered that they were conducted in both early and not early abstainers and that they cannot be allocated to any population severity group.

Studies that were considered as conducted in not early abstainers with H/VH DRL were assigned to the group of high‐severity studies and studies considered as conducted in early abstainers or in L/M DRL patients were assigned to the group of mild‐severity studies (Figure 1).

Because mean values of alcohol consumption and detoxification duration were used to assign studies, it can be argued that these studies may have included a mix of both mild‐severity and high‐severity patients. To address this point, a sensitivity analysis has been performed: the dichotomous population severity factor in the main analysis (mild‐severity vs. high‐severity studies) was replaced by the percentage of high‐severity patients as a continuous variable. The percentage of high‐severity patients assuming independence of both criteria was determined for each study retained by multiplying the percentage of not early abstainers by the percentage of H/VH DRL patients. For instance, for a study with a percentage of not early abstainers of 80% and a percentage of H/VH DRL patients of 70% in the placebo group, the percentage of high‐severity patients is 56% (=80%*70%). The percentage of not early abstainers and of H/VH DRL patients were computed based on the reported mean detoxification duration and mean alcohol consumption in placebo group, respectively, and their related standard deviation and assuming a normal distribution. To assess the possible effect of the probability density function, a further sensitivity analysis using a lognormal distribution of alcohol use and abstinence duration was performed. Additional information on the above methods is available in the Supplementary Material.

Since studies with a mean abstinence duration before treatment initiation between 11 and 17 days were excluded from the sample used in the primary analysis, a sensitivity analysis was conducted adding these studies to the analyzed sample of RCTs (“extended sample”) and investigating the effect of the percentage of high‐severity patients and treatment duration on the placebo response in abstinence rate.

The risk of bias assessment for RCTs in this review was performed using the criteria recommended by the Cochrane Handbook (Higgins et al., 2020): sequence generation; allocation concealment; completeness of outcome data; selective reporting; other possible bias, such as similarity of patients in the groups; blinding of patients, providers, and of subjective outcomes (more information is provided in Supplementary Methods).

### Statistical analyses

The primary outcome for this study was the abstinence rate. The effect of the potential predictors of the abstinence rate in the placebo groups was analyzed by hierarchical multiple meta‐regressions on study level (Harrer et al., 2019; Higgins et al., 2020).

In the main analysis, the two covariates associated with placebo response differences in previous research were included into the meta‐regression model: (1) the dichotomous subpopulation variable as defined above (mild severity vs. high severity), and (2) the intended duration of treatment. In the sensitivity analyses, the two covariates included in the meta‐regression model were as follows: the continuous variable defining the percentage of high‐severity patients and the intended duration of treatment.

A secondary meta‐regression analysis was conducted including the two original explanatory variables (subpopulation and treatment duration) and a new set of independent variables composed of any other baseline patient characteristics reported in retained studies to adjust the effect of factors of interest for potential confounding factors. In order to have enough data for the meta‐regression to be sensitive, only baseline characteristics reported in at least 10 studies were retained for this analysis (Borenstein et al., 2010; Higgins et al., 2020).

The effects of (1) mean alcohol consumption at baseline (in g/day) and treatment duration, (2) mean abstinence duration before treatment initiation (in days) and treatment duration, and (3) mean alcohol consumption at baseline, mean abstinence duration before treatment initiation, and treatment duration on the abstinence rate were also investigated as secondary analyses.

Statistical significance was set at *p* < 0.05. The principal statistical software used was STATA 14 (StataCorp, 2015).

## RESULTS

### Study selection and main characteristics of study populations

**FIGURE 2:**
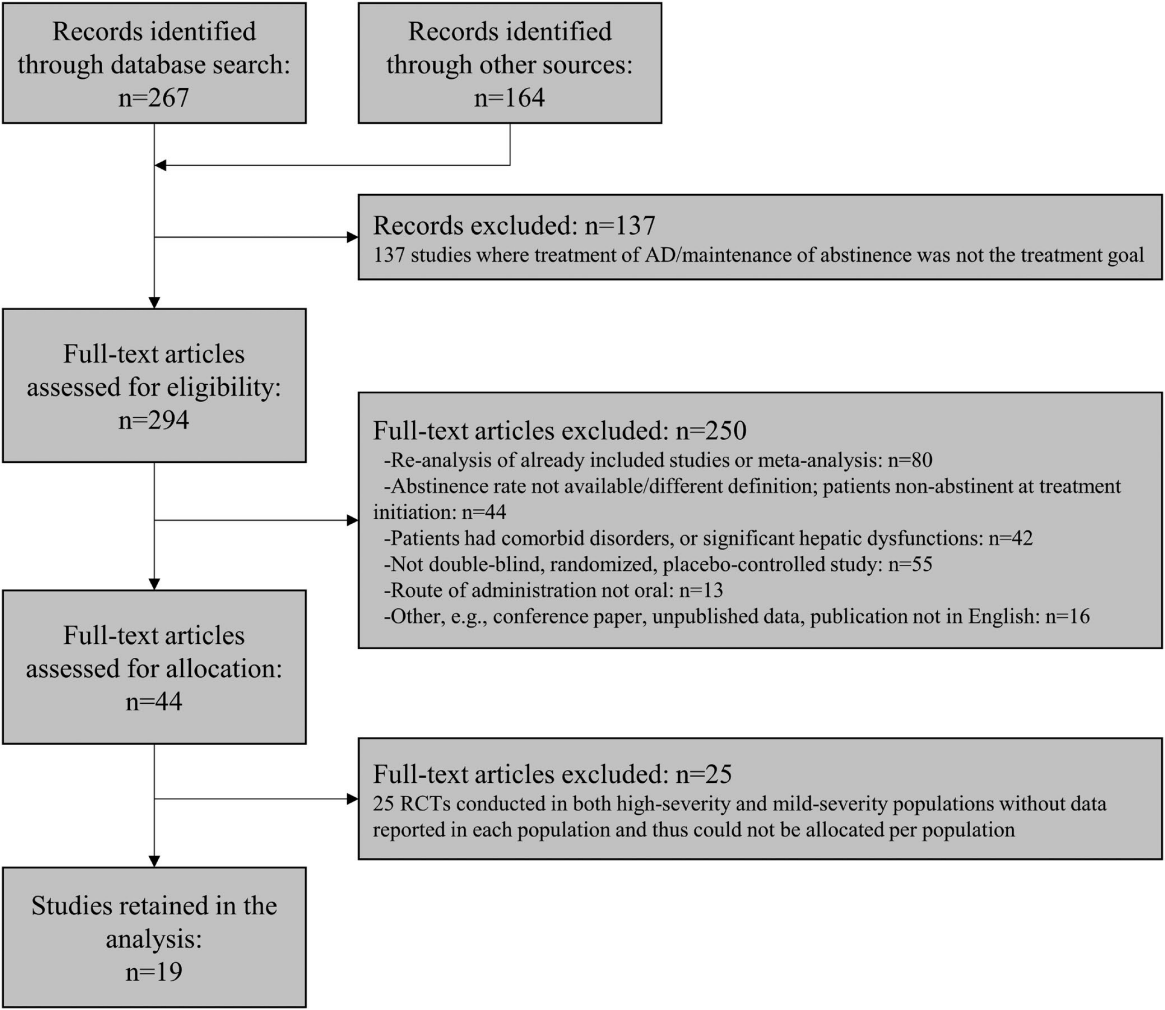
Flow diagram of study selection

In total, 431 articles were screened, 44 fulfilled the selection criteria, and 387 were excluded. The main reasons for exclusion were the following: maintenance of abstinence not the treatment goal (*n* = 137) and reanalysis of already included studies or meta‐analyses (*n* = 80; Figure 2). A list of screened studies and reasons for exclusion is provided in Table S5. Of the 44 studies that fulfilled all selection criteria, 25 were excluded from the analyses because the reported data did not allow assignment of the study to one of the two predefined RCT groups (articles did not report data for each population or data to determine in which population the study was conducted). Among these 25 studies, one reported a mean abstinence duration between 11 and 17 days before treatment initiation (Müller et al., 2015). As a result, 19 RCTs, with 1996 placebo‐treated patients, were assigned to one of the two predefined RCT groups and were thus included in the analyses (see list in Table S4).

The mild‐severity population group consisted of eight studies totaling 920 placebo‐treated patients. The high‐severity population group consisted of 11 studies totaling 1076 placebo‐treated patients.

Table 1 shows the main study population characteristics. Mean age at baseline and the percentage of men were reported in 19 and 18 studies, respectively, and were similar in the two subpopulations. Other baseline characteristics such as mean Alcohol Dependence Scale (Skinner & Horn, 1984) scores were available in less than 10 studies and were thus not included as independent variables in the meta‐regression analysis.

**TABLE 1:** Descriptive statistics and main characteristics of placebo groups in retained studies

Characteristics	Statistical parameters	Overall	Mild‐severity population	High‐severity population
Retained studies	N studies	19	8	11
Sample size	N patients total	1996	920	1076
	Mean (SD)	105.1 (84.6)	115.0 (53.4)	97.8 (103.7)
	Min; Max	8; 392	8; 177	19; 392
Treatment duration (in months)	Mean^a^ (SD)	4.7 (3.0)	6.9 (3.4)	3.2 (1.3)
	Min; Max	1.0; 12.0	3.0; 12.0	1.0; 6.0
	Median	4.0	6.0	3.0
	First and third quartile	3.0; 6.0	5.5; 7.5	3.0; 3.5
Mean age of patients (in years)	Mean^a^ (SD)	45.0 (3.3)	44.4 (4.0)	45.4 (2.9)
	Min; Max	40.5; 53.1	40.5; 53.1	40.6; 49.8
	Median	44.3	43.4	44.3
	First and third quartile	42.5; 46.9	41.9; 45.2	43.6; 47.5
% of male	Mean^a^ (SD)	78.5 (13.9)	78.7 (6.0)	78.4 (18.3)
	Min; Max	42.9; 100	67.8; 88.4	42.9; 100
	Median	78.4	78.7	78.4
	First and third quartile	70.8; 87.1	76.5; 80.9	67.6; 94.0
Mean alcohol consumption at baseline (in g/day)	Mean^a^ (SD)	136.4 (59.7)	155.0 (43.9)	130.8 (64.5)
	Min; Max	75.7; 288.4	106.5; 192	75.7; 288.4
	Median	120.5	166.5	113.1
	First and third quartile	94.6; 166.5	136.5; 179.3	92.7; 128.6
Mean abstinence duration before treatment (in days)	Mean^a^ (SD)	15.4 (15.6)	28.3 (16.4)	5.7 (3.3)
	Min; Max	1.0; 60.0	17.2; 60.0	1.0; 11.0
	Median	10.0	22.8	5.3
	First and third quartile	4.9; 18.9	17.8; 29.0	3.7; 7.5
% of H/VH DRL^b^	Mean^a^ (SD)	83.8 (10.9)	89.4 (9.8)	81.2 (10.9)
	Min; Max	63.8; 100.0	74.0; 100.0	63.8; 100.0
	Median	83.7	89.6	82.0
	First and third quartile	77.6; 90.6	88.3; 95.0	74.6; 87.3
% of not early abstainers^c^	Mean^a^ (SD)	63.5 (43.7)	15.0 (19.2)	97.3 (6.3)
	Min; Max	0.0; 100.0	0.0; 42.8	79.8; 100.0
	Median	95	0.0	100
	First and third quartile	27.9; 100	0.0; 31.3	99.6; 100.0
% of high severity patients^d^	Mean^a^ (SD)	52.8 (37.0)	13.2 (17.4)	80.6 (12.2)
	Min; Max	0.0; 100.0	0.0; 40.6	60.6; 100.0
	Median	65.4	0.0	82.6
	First and third quartile	20.6; 83.2	0.0; 25.8	72.4; 88.7
Abstinence rate (in %)	Mean^a^ (SD)	22.0 (13.2)	29.1 (16.0)	16.8 (8.1)
	Min; Max	4.1; 50.6	9.7; 50.6	4.1; 31.4
	Median	18.8	27.3	16.1
	First and third quartile	12.9; 29.6	18.6; 39.5	12.5; 19.9

^a^ Unweighted estimate.

^b^ Based on reported mean alcohol consumption values at baseline and related standard deviations and assuming a normal distribution.

^c^ Based on inclusion/exclusion criteria or reported mean detoxification period duration values and related standard deviations and assuming a normal distribution.

^d^ Determined by applying the % of not early abstainers to the % of H/VH DRL patients.

The mean (min, max) percentage of high‐severity patients in placebo group was 81% (61%, 100%) in the group of studies assigned to the high‐severity population and 13% (0%, 41%) in the group of studies assigned to the mild‐severity population, indicating that the assignment of RCTs to each population allowed to distinguish RCTs mainly or exclusively conducted in high‐severity patients from RCTs mainly or exclusively conducted in mild‐severity population. The overall percentage of H/VH DRL patients and the mean alcohol consumption at baseline was high and paradoxically slightly larger in the mild population indicating that all retained RCTs included mainly H/VH DRL patients who were either severe (not early abstainers) or mild (early abstainers). Thus, the assignment of studies to the mild‐severity versus the high‐severity group was mainly driven by the abstinence duration before treatment initiation. The mean percentage of not early abstainers was 15% in the group of mild‐severity studies and 97% in the high‐severity studies (Table 1). The mean (min, max) treatment duration was 3.2 (1.0, 6.0) months in the group of studies assigned to the high‐severity population and 6.9 (3.0, 12.0) months in the group of studies assigned to the mild‐severity population.

Results of the bias evaluation showed a low risk of bias in almost all studies for blinding of participants and personnel, incomplete outcome data, selective reporting, and other types of bias. All studies were randomized, and the risk regarding the random sequence generation was judged to be low in 9 RCTs and unclear in the remaining studies. The risk on allocation concealment was judged unclear for most of studies.

Additional information on baseline characteristics and the risk of bias assessment is available in Supplementary Material.

### Relationship of population severity and treatment duration with abstinence rate

Descriptive statistics show that the abstinence rate (16.8% vs. 29.1%) and between‐study variability in abstinence (interquartile range: 7.4% vs. 20.9%) are lower in the high‐severity than in the mild‐severity studies (Table 1).

**FIGURE 3:**
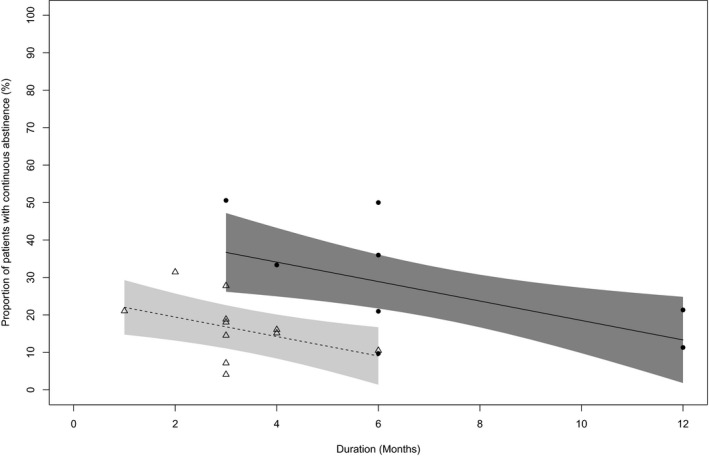
Relationship between abstinence rate and treatment duration in high‐severity population and mild‐severity population (meta‐regression). Circles indicate studies in mild‐severity population, and the line shows the regression with 95% confidence band. The triangles show studies in high‐severity population and the dotted line shows the regression with 95% confidence band

In the primary meta‐regression analysis, the effects of both population severity and treatment duration were significant (*p* = 0.004 and *p* = 0.017, respectively), indicating that the placebo response in abstinence rate was significantly dependent upon population severity and treatment duration (Table 2). For a 3‐month treatment duration, the predicted value of the placebo response in abstinence rate was 16.8% in the high‐severity population and 36.7% in the mild‐severity population: a significant and clinically relevant difference of 19.9% (Table 3). Likewise, the predicted value of the placebo response in abstinence rate was 9.0% in the high‐severity population and 28.9% in the mild‐severity population for a 6‐month treatment duration (Table 3). After adjustment for population severity, the placebo response in abstinence rate decreased by 2.6% per month of treatment, e.g., the longer the treatment duration, the lower the placebo response (Figure 3; Table S3). The adjusted coefficient of determination (*R* ^2^) was 0.39.

**TABLE 2:** Results of meta‐regression models with abstinence rate as the dependent outcome

Analysis	Terms	Estimate	*p* value	Adjusted^a^ *R* ^2^	Heterogeneity
*Primary analysis*					
Main analysis	Tx Duration	−0.0256	*p* = 0.017	$R_{\text{adj}.}^{2}$ = 0.39	*I* ^2^ = 0.84
	Pop. severity	−0.1987	*p* = 0.004		τ^2^ = 0.007
Sensitivity analysis: % of high‐severity patients					
Normal distribution	Tx Duration	−0.0252	*p* = 0.013	$R_{\text{adj}.}^{2}$ = 0.53	*I* ^2^ = 0.82
	% Severe	−0.3153	*p* = 0.001		τ^2^ = 0.007
Lognormal distribution	Tx Duration	−0.0236	*p* = 0.012	$R_{\text{adj}.}^{2}$ = 0.58	*I* ^2^ = 0.80
	% Severe	−0.3023	*p* = 0.001		τ^2^ = 0.006
Extended set of studies^b^	Tx Duration	−0.0249	*p* = 0.012	$R_{\text{adj}.}^{2}$ = 0.53	*I* ^2^ = 0.81
	% Severe	−0.3150	*p* = 0.001		τ^2^ = 0.006
*Secondary analyses*					
Age and % of males	Tx Duration	−0.0265	*p* = 0.027	$R_{\text{adj}.}^{2}$ = 0.32	*I* ^2^ = 0.86
	Pop. severity	−0.1978	*p* = 0.008		τ^2^ = 0.009
	Age	−0.0000	*p* = 0.998		
	% Male	0.1643	*p* = 0.368		
Alcohol consumption at baseline	Tx Duration	−0.0162	*p* = 0.280	$R_{\text{adj}.}^{2}$ = −0.06	*I* ^2^ = 0.88
	Consumption	0.0004	*p* = 0.534		τ^2^ = 0.014
Abstinence duration before treatment initiation	Tx Duration	−0.0207	*p* = 0.138	$R_{\text{adj}.}^{2}$ = 0.26	*I* ^2^ = 0.85
	Abs Duration	0.0054	*p* = 0.041		τ^2^ = 0.011
Abstinence duration and alcohol consumption	Tx duration	−0.0377	*p* = 0.051	$R_{\text{adj}.}^{2}$ = 0.39	*I* ^2^ = 0.84
	Abs duration	0.0194	*p* = 0.044		τ^2^ = 0.010
	Consumption	0.0005	*p* = 0.440		

Tx: treatment; Pop: population; Abs Duration: mean abstinence duration before treatment initiation; Consumption: mean alcohol consumption at baseline.

^a^ The adjusted *R* ^2^ adjusts for the number of terms in the model.

^b^ Assuming a normal distribution. The extended set of studies included 20 RCTs: the 19 studies retained in the main analysis and the study with a mean abstinence duration before treatment initiation between 11 and 17 days: Müller et al. (2015). The percentage of high‐severity patient in this study was estimated at 61%, the retained treatment duration was 4 months, the reported abstinence rate was 14.3%, and the placebo group included 28 patients.

**TABLE 3:** Predicted values (95% CI) of the placebo response in abstinence rate

Predicted values	Month 3	Month 6
Mild‐severity population		
Main analysis	36.7% (26.2; 47.2)	28.9% (21.7; 36.1)
Sensitivity analysis—normal distribution^a^	42.1% (30.4; 53.8)	34.5% (25.7; 43.4)
Sensitivity analysis—lognormal distribution^a^	42.3% (31.3; 53.4)	35.3% (26.7; 43.9)
High‐severity population		
Main analysis	16.8% (11.1; 22.6)	9.0% (1.4; 16.7)
Sensitivity analysis—normal distribution^b^	10.6% (3.2; 18.0)	3.0% (−6.4; 12.4)
Sensitivity analysis—lognormal distribution^b^	12.1% (5.7; 18.6)	5.0% (−2.9; 13.0)

^a^ Predicted estimates for a % of high‐severity patients of 0%.

^b^ Predicted estimates for a % of high‐severity patients of 100%

In the sensitivity analyses, the effects of the percentage of high‐severity patients and of treatment duration factors were also statistically significant with similar *p* values but with a higher percentage of variance explained than in the main analysis and with no relevant difference in variance explained between the model assuming normal and lognormal distributions of alcohol use and abstinence duration: adjusted *R* ^2^ = 0.53 assuming normal distributions and adjusted *R* ^2^ = 0.58 assuming lognormal distributions. Similar estimates and *p* values of the effects of the percentage of high‐severity patients and of treatment duration factors were observed when the study with a mean abstinence duration between 11 and 17 days before treatment initiation was included in the sample analyzed in the meta‐regression model (Table 2; Table S3).

In the secondary analysis with the mean age at baseline and the percentage of males included in the meta‐regression analysis, results showed no significant effect of these factors on the placebo response whereas the effect of population severity and of treatment duration remained significant (*p* = 0.008 and *p* = 0.027, respectively). The mean alcohol consumption at baseline adjusted for treatment duration was not a significant predictor of the placebo response (*p* = 0.534), whereas the mean abstinence duration before treatment initiation adjusted for treatment duration had a significant effect on the placebo response (*p* = 0.041). However, the variance explained by this model (adjusted *R* ^2^ = 0.26) was lower than in the primary analysis. In the model with the mean abstinence duration before treatment initiation, the mean alcohol consumption at baseline, and treatment duration as independent variables, only the abstinence duration before treatment initiation showed a significant effect on the placebo response (*p* = 0.044), but the variance explained by this model was similar to the primary analysis (adjusted *R* ^2^ = 0.39; see Table 2; Table S3).

## DISCUSSION

The effect size of pharmacological interventions for the treatment of AD is generally rather modest and is negatively correlated with the placebo response rate in RCTs (Jonas et al., 2014; Litten et al., 2013). However, understanding the nature of the placebo effect in RCTs for AD remains poor and a better characterization of factors that predict placebo response is warranted. Indeed, placebo effects are powerful and common in neuropsychiatric disorders and in clinical practice in general (Colloca & Barsky, 2020).

Here, we studied population severity and treatment duration as two potential drivers of the placebo response in these studies. The population severity is categorical variable with two categories which have been defined in literature with respect to their effect on placebo response and treatment effect. It distinguishes heavy drinkers without “spontaneous improvement” prior to treatment initiation (high severity) from other patients (mild severity; van den Brink et al., 2018).

In our meta‐regression analysis of 19 RCTs with 1996 placebo‐treated AD patients, placebo response in abstinence rate was significantly lower in the high‐severity studies than in the mild‐severity studies. The significant decrease by 19.9% in points for the placebo response in abstinence rate in the high‐severity compared with the low‐severity studies is clinically meaningful. These results are in line with previous subgroup analyses of single RCTs for AD where early abstainers/reducers and L/M DRL patients showed a higher placebo response (and a lower treatment effect) than H/VH DRL patients not early abstainer/reducers (Gual et al., 2013; Gueorguieva et al., 2011, 2012; Mann et al., 2016; van den Brink et al., 2018, 2013, 2014). In addition, spontaneous improvement prior to randomization is a recognized predictor of higher placebo response in other therapeutic areas such as depression, anxiety, angina, dyslipidemia, hypertension (Doering et al., 2014; Sonawalla & Rosenbaum, 2002; US Food & Drug Administration, 2019).

These findings were supported in the current meta‐regression analysis by results from a sensitivity analysis using the percentage of high‐severity patients in the studies as a predictor of placebo response. Moreover, the variance explained was higher in the sensitivity analysis than in the primary analysis. This may be partly explained by the use of a continuous predictive variable which considers that certain studies had a mixed population of mild‐severity and high‐severity patients. Almost identical results were obtained in the sensitivity analysis using the extended sample which included 20 RCTs: the 19 retained RCTs as well as the Müller et al. (2015) study reporting a mean abstinence duration before treatment initiation between 11 and 17 days. These data indicate that the placebo response in the Müller et al. (2015) study was consistent with the modeling estimate based on the 19 RCTs, which strengthens the results of the primary analysis. Interestingly, the reported placebo response in abstinence rate in Müller et al. (2015) is 14.3% when the predicted estimates of the placebo response for this study are 20.3% and 16.1% in the models assuming a normal and a lognormal distribution, respectively, and using the main sample of studies (i.e., 19 RCTs). Therefore, and since it also showed the highest variance explained (adjusted *R* ^2^ = 0.58), the model using the percentage of high‐severity patients adjusted for treatment duration and assuming a lognormal distribution of the alcohol consumption at baseline and of the abstinence duration pretreatment may provide better predicted estimates of the placebo response.

In secondary analyses, the placebo response was associated with the mean abstinence duration before treatment initiation but not with the mean alcohol consumption at baseline. In our meta‐regression, the population severity effect was mainly driven by the “early abstainer” factor as opposed to previous subgroup analyses of RCTs where population severity was driven exclusively by baseline DRL (van den Brink et al., 2018) or by both baseline DRL and early abstainer/reducer (Gual et al., 2013; Gueorguieva et al., 2011, 2012; Mann et al., 2016; van den Brink et al., 2013, 2014). This difference in the explanatory power of each factor can be explained as follows: in the current meta‐regression analysis, the percentage of early abstainers varied widely, whereas the baseline DRL was very similar across studies (similar percentage of H/VH DRL). Thus, this DRL factor was almost a constant in the present analysis (that cannot explain variance of the response), whereas the early abstainer factor showed a large range of variation (and can explain variance of the response). Consequently, in the current meta‐analysis, the population severity effect was mainly driven by the early abstainer factor. In another study, the opposite was the case: big variation in population severity and small or no variation in early abstainers and thus the population severity effect was driven by the baseline DRL factor (van den Brink et al., 2018). Finally, in RCTs where the population severity effect was driven by both baseline DRL and early abstainer/reducer, both factors showed large variation (Gual et al., 2013; Gueorguieva et al., 2011, 2012; van den Brink et al., 2013, 2014). Thus, the contribution of each factor (baseline DRL vs. early abstinence) in the population severity effect on the placebo response appears to be dependent on sample and study design.

The current meta‐regression analysis reconciles the seemingly conflicting results related to the effect of treatment duration on abstinence in the placebo group between patient‐level and study‐level analyses. At study level and in the current analysis, treatment duration adjusted for population severity was a predictor of the placebo response as consistently shown by others at the patient level in single RCTs (Anton et al., 1999, 2005; Baltieri & Andrade, 2003; Baltieri et al., 2008; Chick et al., 2000; Kiefer et al., 2003; Pelc et al., 1997; Volpicelli et al., 1997). However, in a previous meta‐analysis of 51 RCTs, the placebo response at study level was not dependent on the unadjusted treatment duration (Litten et al., 2013). In the current meta‐regression analysis, the placebo response was also not dependent on treatment duration when the latter was adjusted for the mean alcohol consumption at baseline and/or the mean abstinence duration before treatment initiation. These results suggest that, at study level, treatment duration should be adjusted for population severity to show an effect on abstinence in the placebo group.

The effects of mean age at baseline and percentage of males on the placebo response in abstinence rate were not significant and support prior results for percentage of males in another meta‐analysis (Litten et al., 2013). However, in this previous meta‐analysis, mean age was associated with the placebo response in the percentage of days abstinent in naltrexone RCTs but not in acamprosate studies (Litten et al., 2013).

Results of our meta‐regression also showed a decrease of between‐study variability in response rates in the placebo group. Consequently, power calculation of future RCTs should be more reliable because the expected treatment effect is less random. This approach should improve assay sensitivity in the detection of true‐positive treatment effects (Litten et al., 2012). Nevertheless, the complex nature of the placebo response was not fully explained by population severity and the treatment duration, and other factors must be explored at the patient level to further reduce placebo response variability. Since abstinence was determined using patient's self‐reported alcohol consumption, some of the unexplained variance in the placebo response may also be due to the inaccuracy of self‐reported measures of alcohol consumption (de Bejczy et al., 2015). In addition, the retained trials may have included some patients for whom the drinking goal was not abstinence (but reduced drinking) which may have had an effect on the placebo response and this mismatch of treatment goals may explain a portion of the residual unexplained variance (Bujarski et al., 2013; DeMartini et al., 2014).

The number of studies conducted in the mild‐severity and the high‐severity population was rather well balanced with eight and 11 studies and about 1000 patients in each subpopulation. Such balance provides better power to detect the effect of study factors. However, the predicted values for the placebo response are limited by a treatment duration of only 6 months in the enriched (e.g., high‐severity) population, because there were no studies with a longer treatment duration that also met all study inclusion criteria.

Results of risk of bias evaluation showed a low or unclear risk of bias for almost all criteria and all studies.

Overall, our results call for the reevaluation of large trials conducted in unselected study populations and reanalysis of the data considering baseline DRL and abstinence duration prior to treatment. This approach was recently applied to an RCT with sodium oxybate (van den Brink et al., 2018). In the latter study, the abstinence rate in the placebo arm at the end of the 3‐month treatment period was 15% in the high‐severity population compared with 40% in the mild‐severity population which is consistent with our modeling estimates. This post hoc finding strengthens the conclusions of the current systematic study on the population enrichment strategy. The here proposed enrichment strategy could be practically implemented by enrolling only H/VH DRL patients and by applying a treatment‐free run‐in period of at least 2 weeks to exclude patients with a mild disorder and/or spontaneous improvement.

A potential drawback of applying enrichment strategies is a decrease of external validity/generalizability through exclusion of a specific part of the patient population (Leber & Davis, 1998). However, the lower generalizability of the findings from the here proposed enrichment strategy is justified by the fact that this group of AD patients is responsible for the majority of AD attributable burden (Rehm et al., 2018). Furthermore, the limitation of reduced generalizability can be handled with a restriction of the indication to the high‐severity population for medicinal products which have demonstrated efficacy and a positive benefit risk in the enriched population. In this respect and while the clinical development included both populations, nalmefene efficacy in the treatment of AD was established in the high‐severity population only and the compound was consecutively approved by the EMA in this restricted population which excludes L/M DRL patients and early abstainers/reducers (European Medicines Agency, 2012). We, therefore, are in favor of the use of population enrichment strategies to improve assay sensitivity in trials with alcohol use disorder patients. In conclusion, the present work supports the use of population enrichment approaches to improve assay sensitivity in clinical trials with AD patients. The goal of such an approach is to enroll only patients with the highest probability to benefit from pharmacological treatments, thus improving our ability to develop novel precision medicines.

## CONFLICT OF INTEREST

Giovanni Addolorato served as a consultant for Ortho‐McNeil Janssen Scientific Affairs, LLC, and D&A Pharma, and was paid for his consulting services. He has received lecture fees from D&A Pharma. Henri‐Jean Aubin reports being member of advisory boards or DSMB for Bioprojet, and Ethypharm and has received sponsorship to attend scientific meetings, speaker honoraria, or consultancy fees from Bioprojet, D&A Pharma, Ethypharm, Kinnov Pharmaceuticals, and Lundbeck. He is also member of the American Society of Clinical Psychopharmacology's Alcohol Clinical Trials Initiative (ACTIVE), which was supported in the last 3 years by Alkermes, Amygdala Neurosciences, Arbor Pharmaceuticals, Indivior, Lundbeck, Mitsubishi, and Otsuka. Rolland Benjamin received fees from Ethypharm and Lundbeck. David Nutt reports personal fees from D&A Pharma and Lundbeck and is a director of Alcarelle. Wim van den Brink reported personal fees from D&A Pharma, Kinnov Therapeutics, Bioproject, Lundbeck, Novartis, Indivior, Angelini, Mundipharma, Takeda, and Opiant Inc. Maurice Dematteis has provided expert advice to Bouchara‐Recordati Laboratories, Camurus, Indivior, Lundbeck, and D&APharma and received fees for lectures from Bouchara‐Recordati Laboratories, Lundbeck, Camurus, and Indivior. Antony Gual reports grants from Novartis and D&A Pharma. Lorenzo Leggio is a U.S. federal employee and is supported by the NIDA and NIAAA intramural research programs. He has also received royalties from Routledge Press (textbook) and honoraria from the UK Medical Council on Alcoholism (Editor‐in‐Chief for Alcohol and Alcoholism). Jürgen Rehm reported personal fees from D&A Pharma and Lundbeck. Rainer Spanagel reported grants from Horizon 2020 program, Era‐NET NEURON, BMBF, Deutsche Forschungsgemeinschaft (DFG) and personal fees from EMCCDA and D&A Pharma during the conduct of the study. Bruno Scherrer reported fees from D&A pharma, DNDI, HRA Pharma, and other pharmaceutical organizations. JG is employed by D&A Pharma which was one sponsor of this study. RP and QR were employed by D&A Pharma. The other funders had no role in the design and conduct of the study; collection, management, analysis, and interpretation of the data; preparation, review, or approval of the manuscript; and decision to submit the manuscript for publication.

## AUTHOR CONTRIBUTIONS

BS, JG, and RS designed the analysis strategy. Literature search was performed by JG, RP, QR, and RS. Statistical analysis was performed by BS, QR, JG, and RP. JG, BS, and RS wrote the manuscript and all authors contributed to and have approved the final manuscript.

## Supporting information

Supinfo S1
