# Supplementary material for: Baseline severity and the prediction of placebo response in clinical trials for alcohol dependence: A meta‐regression analysis to develop an enrichment strategy
Source: Alcohol Clin Exp Res. 2021 Aug 21;45(9):1722–34. doi: 10.1111/acer.14670 (PMC9291112; doi:10.1111/acer.14670)
Supplement: Supplementary file 1 — Supinfo S1 [file ACER-45-1722-s001.docx]

Supplementary Information

**Search terms selected for systematic literature search**

1) Alcohol

2) Dependence

3) Abstinence

4) 1 and 2 and 3

5) Nalmefene

6) Baclofen

7) Disulfiram

8) Naltrexone

9) Sodium oxybate

10) Gabapentin

11) Topiramate

12) Acamprosate

13) Ondansetron

14) Quetiapine

15) Kappa

16) Zoliynamide

17) ABT-436

18) Prazosin

19) Doxazosin

20) Varenicline

21) Aripiprazole

22) 5 or 6 or 7 or 8 or 9 or 10 or 11 or 12 or 13 or 14 or 15 or 16 or 17 or 18 or 19 or 20 or 21;

23) Double-blind

24) Controlled

25) 23 or 24

26) Placebo

27) 4 and 22 and 25 and 26

Table S1: WHO classification of pure alcohol consumption per day in relation to risk of acute problems

| **Drinking Risk Level (DRL)** | **Male** | **Female** |
| --- | --- | --- |
| Low DRL | 1 to 40g | 1 to 20g |
| Medium DRL | 41 to 60g | 21 to 40g |
| High DRL | 61 to 100g | 41 to 60g |
| Very High DRL | 101+g | 61+g |

### **Methods of allocation of studies in each population**

1. Main analysis: binary allocation of studies in two study populations: high-severity vs mild-severity population. Population severity was based on the two criteria defined in the literature: alcohol consumption at baseline and abstinence duration prior to treatment (see Figure 1).

The first criterion is based on WHO DRL to categorize patients depending on their mean alcohol consumption (in grams of pure alcohol per day) at baseline. If the mean alcohol consumption at baseline in placebo group was lower than the Medium DRL threshold the study was considered as being conducted in the Low or Medium (L/M) DRL population. The Medium DRL threshold was determined for each trial using the study proportion of men and women. For instance, if the study population was composed of 75% men and 25% women the Medium DRL threshold was 55g (75%*60g+25%*40g). If only the mean number of standard drinks at baseline in the placebo group was reported, then the conversion to grams was performed using the following country-specific standard drinking units: South Korea 8g; Australia, Belgium, France 10g; Italy 12g; United States of America 14g; Germany 15g (WHO, 2018). Studies conducted in L/M DRL population were allocated to the mild-severity population regardless of the abstinence duration prior to treatment.

For RCTs not categorized as L/M DRL studies, a second criterion linked to the abstinence duration before treatment initiation was applied, which allowed to distinguish not early abstainers from other patients. If the inclusion/exclusion criteria specified a detoxification period of less than 14 days prior to treatment initiation, the study was considered to be conducted in not early abstainers. Conversely, studies with inclusion/exclusion criteria specifying a detoxification period longer than 14 days were considered to be conducted in early abstainers. The mean detoxification duration was used in case it was not possible to classify the study based on the inclusion/exclusion criteria. Studies with a mean detoxification duration of less than 11 days were considered as being conducted in not early abstainers. Conversely, studies with a mean detoxification duration longer than 17 days were considered as conducted in early abstainers. Studies with a mean detoxification period between 11 and 17 days were excluded as it was too close from the 14 days threshold and thus, we considered that they were conducted in both early and not early abstainers and that they cannot be allocated to any population severity group.

Studies that were considered as conducted in not early abstainers with H/VH DRL were assigned to the group of high-severity studies and studies considered as conducted in early abstainers or in L/M DRL patients were assigned to the group of mild-severity studies.

2. Sensitivity analysis: study allocation based on the % of high-severity patients enrolled in each study

Studies’ allocation method in the above main analysis used the mean alcohol consumption at baseline and/or the mean detoxification duration in placebo group for all studies. Because mean values were used, it can be argued that these studies may have not been conducted in studies with exclusively mild-severity or high-severity patients. To address this point, a sensitivity analysis has been carried out: the population severity factor which was a dichotomous variable in the main analysis (mild-severity or high-severity) is replaced in the meta-regression model by the percentage of high-severity patients enrolled in the placebo group (which is a continuous variable).

Studies with inclusion/exclusion criteria specifying a detoxification period longer than 14 days were considered as having no (i.e. 0%) high-severity patients in placebo group regardless of the DRL at baseline because the study was exclusively conducted in early abstainers and thus in the mild severity population.

For studies with inclusion/exclusion criteria specifying a detoxification period of less than 14 days, the percentage of H/VH DRL and thus of high-severity patients was determined based on the reported mean alcohol consumption at baseline in placebo group and its related standard deviation and assuming a probability density function following a normal distribution. The percentage of high-severity patients represented the percentage of the normal distribution above the Medium DRL threshold.

For the remaining studies, the percentage of not early abstainers was obtained based on the reported mean detoxification duration in placebo group and its related standard deviation and assuming a normal distribution. The percentage of not early abstainers represented the percentage of the normal distribution below the 14 days detoxification duration threshold. The percentage of high-severity patients for these studies was determined by applying the percentage of not early abstainers to the percentage of H/VH DRL patients. For instance, for a study with a percentage of not early abstainer of 80% and a percentage of H/VH DRL patients of 70% in the placebo group, the percentage of high-severity patients is 56% (=80%*70%).

To assess the possible effect of the probability density function on the percentage of H/VH DRL and the percentage of not early abstainers, a further sensitivity analysis using a lognormal distribution of alcohol use and abstinence duration was performed.

Table S2: Descriptive statistics and main characteristics of populations

| **Characteristics** | **Statistical parameters** | **Overall** | **Mild-severity population** | **High-severity population** |
| --- | --- | --- | --- | --- |
| Available studies | N studies | 19 | 8 | 11 |
| Sample size (PBO) | N patients total | 1 996 | 920 | 1 076 |
|  | Mean (SD) | 105.1 (84.6) | 115.0 (53.4) | 97.8 (103.7) |
|  | Min; Max | 8; 392 | 8; 177 | 19; 392 |
| Treatment duration  (in months) | Mean^*^ (SD) | 4.7 (3.0) | 6.9 (3.4) | 3.2 (1.3) |
|  | Min; Max | 1.0; 12.0 | 3.0; 12.0 | 1.0; 6.0 |
|  | Range across studies | 11.0 | 9.0 | 5.0 |
|  | Median | 4.0 | 6.0 | 3.0 |
|  | 1st and 3rd quartile | 3.0; 6.0 | 5.5; 7.5 | 3.0; 3.5 |
|  | Interquartile range | 3.0 | 2.0 | 0.5 |
| Mean age of patients in PBO group  (in years) | Mean^*^ (SD) | 45.0 (3.3) | 44.4 (4.0) | 45.4 (2.9) |
|  | Min; Max | 40.5; 53.1 | 40.5; 53.1 | 40.6; 49.8 |
|  | Range across studies | 12.6 | 12.6 | 9.2 |
|  | Median | 44.3 | 43.4 | 44.3 |
|  | 1st and 3rd quartile | 42.5; 46.9 | 41.9; 45.2 | 43.6; 47.5 |
|  | Interquartile range | 4.5 | 3.3 | 3.9 |
| % of male in PBO group | Mean^*^ (SD) | 78.5 (13.9) | 78.7 (6.0) | 78.4 (18.3) |
|  | Min; Max | 42.9; 100 | 67.8; 88.4 | 42.9; 100 |
|  | Range across studies | 57.1 | 20.6 | 57.1 |
|  | Median | 78.4 | 78.7 | 78.4 |
|  | 1st and 3rd quartile | 70.8; 87.1 | 76.5; 80.9 | 67.6; 94.0 |
|  | Interquartile range | 16.3 | 4.4 | 26.4 |
| % of patients with H/VH DRL at baseline in PBO group | Mean^*^ (SD) | 83.8 (10.9) | 89.4 (9.8) | 81.2 (10.9) |
|  | Min; Max | 63.8; 100.0 | 74.0; 100.0 | 63.8; 100.0 |
|  | Range across studies | 36.3 | 26.1 | 36.3 |
|  | Median | 83.7 | 89.6 | 82.0 |
|  | 1st and 3rd quartile | 77.6; 90.6 | 88.3; 95.0 | 74.6; 87.3 |
|  | Interquartile range | 13.0 | 6.7 | 12.7 |
| % of patients not early abstainers at baseline in PBO group | Mean^*^ (SD) | 63.5 (43.7) | 15.0 (19.2) | 97.3 (6.3) |
|  | Min; Max | 0.0; 100.0 | 0.0; 42.8 | 79.8; 100.0 |
|  | Range across studies | 100.0 | 42.8 | 20.2 |
|  | Median | 95.0 | 0.0 | 100 |
|  | 1st and 3rd quartile | 27.9; 100.0 | 0.0; 31.3 | 99.6; 100.0 |
|  | Interquartile range | 72.1 | 31.3 | 0.4 |
| % of patients with high severity at baseline in PBO group | Mean^*^ (SD) | 52.8 (37.0) | 13.2 (17.4) | 80.6 (12.2) |
|  | Min; Max | 0.0; 100.0 | 0.0; 40.6 | 60.6; 100.0 |
|  | Range across studies | 100.0 | 40.6 | 39.4 |
|  | Median | 65.4 | 0.0 | 82.6 |
|  | 1st and 3rd quartile | 20.6; 83.2 | 0.0; 25.8 | 72.4; 88.7 |
|  | Interquartile range | 62.5 | 25.8 | 16.3 |
| Abstinence rate in PBO group  (in %) | Mean^*^ (SD) | 22.0 (13.2) | 29.1 (16.0) | 16.8 (8.1) |
|  | Min; Max | 4.1; 50.6 | 9.7; 50.6 | 4.1; 31.4 |
|  | Range across studies | 46.5 | 40.9 | 27.3 |
|  | Median | 18.8 | 27.3 | 16.1 |
|  | 1st and 3rd quartile | 12.9; 29.6 | 18.6; 39.5 | 12.5; 19.9 |
|  | Interquartile range | 16.7 | 20.9 | 7.4 |

*Unweighted estimate

**Table S3: Meta regression analysis with abstinence rate as the dependent outcome (response)**

| **Model terms:** | **Terms** | **Estimate of parameter** | **p value** | **Effect** |
| --- | --- | --- | --- | --- |
| Duration + Population severity | Constant Duration Severity | 0.44481 -0.02597 -0.19865 | p<0.001 p=0.017 p=0.004 | R²_adj._ = 0.39; I²=0.84 tau²=0.007 Decrease of 2.60 points per month of treatment  Decrease of 19.87 points in severe population |
| Duration + Population severity + % of male + Age | Constant Duration Severity % of male Age | 0.32031 -0.02648 -0.19782 0.16432 -0.00002 | p=0.407 p=0.027 p=0.008 p=0.368 p=0.998 | R²_adj._ = 0.32; I²=0.86 tau²=0.009 Decrease of 2.65 points per month of treatment  Decrease of 19.78 points in severe population |
| Duration + % high severity patients^1^ | Constant Duration % severe^1^ | 0.49681 -0.02524 -0.31531 | p<0.001 p=0.013 p=0.001 | R²_adj._ = 0.53; I²=0.82 tau²=0.007 Decrease of 2.52 points per month of treatment  Decrease of 0.32 points per point of % severe |
| Duration + % high severity patients^2^ | Constant Duration % severe^2^ | 0.49403 -0.02355 -0.30227 | p<0.001 p=0.012 p=0.001 | R²_adj._ = 0.58; I²=0.80 tau²=0.006 Decrease of 2.36 points per month of treatment  Decrease of 0.30 points per point of % severe |
| Duration + % high severity patients^3^ | Constant Duration %severe^3^ | 0.49202 -0.02491 -0.31502 | p<0.001 p=0.012 p=0.001 | R²_adj._ = 0.53; I²=0.81 tau²=0.006 Decrease of 2.49 points per month of treatment  Decrease of 0.32 points per point of % severe |
| Duration + alcohol consumption | Constant Duration Alcohol cons. | 0.20592  -0.01624 0.00043 | p=0.064 p=0.280 p=0.534 | R²_adj._ = -0.06; I²=0.88 tau²=0.014 |
| Duration + abstinence duration prior to treatment | Constant Duration Abstinence dur. | 0.00537 -0.02072 0.00537 | p=0.004 p=0.138 p=0.041 | R²_adj._ = 0.26; I²=0.85 tau²=0.011 Increase of 0.54 points per day of abstinence before treatment |
| Duration + alcohol consumption + abstinence duration prior to treatment | Constant Duration Alcohol cons.  Abstinence dur. | 0.11063 -0.03771 0.00053 0.01938 | p=0.284 p=0.051 p=0.440 p=0.044 | R²_adj._ = 0.39; I²=0.84 tau²=0.010 Increase of 1.94 points per day of abstinence before treatment |

^1^: normal distribution

^2^:log-normal distribution

^3^: normal distribution and study sample including the study with mean abstinence duration before randomization between 11 and 17 days

Figure S2: Abstinence rate, treatment duration and percentage of high-severity patients in placebo group in retained studies


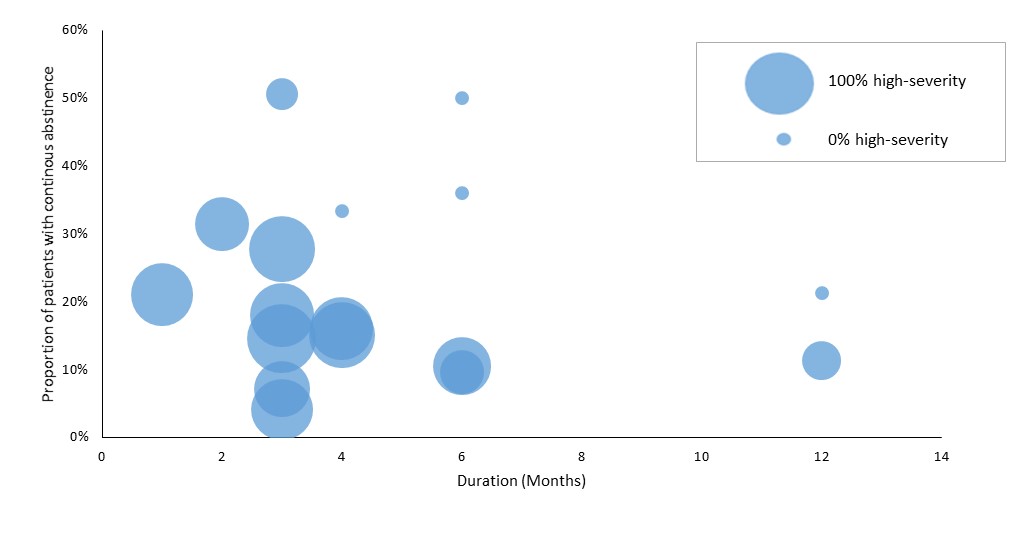


## Table S4: Information on selected studies

| **Study name** | **Author** | **Population** | **% of high-severity patients^1^** | **% of not early abstainers^1^** | **% of H/VH DRL^1^** | **Treatment Duration (months)** | **Abstinence. rate (n/N)** |
| --- | --- | --- | --- | --- | --- | --- | --- |
| Lack of efficacy of naltrexone in the prevention of alcohol relapse: results from a German multicenter study | Gastpar et al. 2002 | Mild | 21% | 28% | 74% | 3 | 44/87 |
| Baseline trajectories of drinking moderate acamprosate and naltrexone effects in the COMBINE study | Gueorguieva et al. 2011 | Mild | 0% | 0% | 88% | 4 | 30/90 |
| Gamma-hydroxybutyric acid induced suppression and prevention of alcohol withdrawal syndrome and relief of craving in alcohol-dependent patients | Di Bello et al. 1995 | Mild | 0% | 0% | 100% | 6 | 4/8 |
| Acamprosate and Prevention of Relapse in Alcoholics | Geerlings et al. 1997 | Mild | 41% | 43% | 95% | 6 | 13/134 |
| Efficacy of Acamprosate for the Treatment of Alcohol Dependence Long After Recovery From Withdrawal Syndrome: A Randomized, Double-Blind, Placebo-Controlled Study Conducted in Japan (Sunrise Study) | Higuchi et al. 2015 | Mild | 0% | 0% | NA | 6 | 59/164 |
| Acamprosate treatment in a long-term community-based alcohol rehabilitation programme | Poldrugo 1997 | Mild | ND | NA | NA | 6 | 26/124 |
| Double-blind randomized multicentre trial of acamprosate in maintaining abstinence from alcohol | Paille et al. 1995 | Mild | 31% | 35% | 90% | 12 | 20/177 |
| Relapse prevention by acamprosate: results from a placebo-controlled study on alcohol dependence | Sass et al. 1996 | Mild | 0% | 0% | NA | 12 | 29/136 |
| Baclofen efficacy in reducing alcohol craving and intake: a preliminary double-blind randomized controlled study | Addolorato et al. 2002 | High-severity | 82% | 100% | 82% | 1 | 4/19 |
| Acamprosate in Korean alcohol-dependent patients: a multi-centre, randomized, double-blind, placebo-controlled study | Namkoong et al. 2003 | High-severity | 61% | 95% | 64% | 2 | 22/70 |
| Comparing topiramate with naltrexone in the treatment of alcohol dependence* | Baltieri et al. 2008 | High-severity | 90% | 100% | 90% | 3 | 15/54 |
| Gabapentin Treatment for Alcohol Dependence: A Randomized Clinical Trial | Mason et al. 2014 | High-severity | 79% | 100% | 79% | 3 | 2/49 |
| Naltrexone versus acamprosate in the treatment of alcohol dependence: a multi-centre, randomized, double-blind, placebo-controlled trial | Morley et al. 2006 | High-severity | 84% | 100% | 84% | 3 | 11/61 |
| Naltrexone for alcohol dependence: a randomized controlled trial | Morris et al. 2001 | High-severity | 65% | 80% | 82% | 3 | 4/56 |
| A multicentre, randomised, double-blind, placebo-controlled trial of naltrexone in the treatment of alcohol dependence or abuse | Chick 2000 | High-severity | ND | NA | 67% | 3 | 16/85 |
| Efficacy and safety of acamprosate in the treatment of detoxified alcohol-dependent patients. A 90-day placebo-controlled dose-finding study. | Pelc et al. 1997 | High-severity | 100% | 100% | 100% | 3 | 9/62 |
| A Double-Blind, Placebo-Controlled Trial Assessing the Efficacy of Levetiracetam Extended-Release in Very Heavy Drinking Alcohol-Dependent Patients | Fertig et al. 2012 | High-severity | 91% | 100% | 91% | 4 | 10/66 |
| Baseline trajectories of drinking moderate acamprosate and naltrexone effects in the COMBINE study | Gueorguieva et al. 2011 | High-severity | 83% | 99% | 83% | 4 | 63/392 |
| A randomized, placebo-controlled study of high-dose baclofen in alcohol-dependent Patients—The ALPADIR study | Reynaud et al. 2017 | High-severity | 70% | 100% | 70% | 6 | 17/162 |

NA: not available; reported data were insufficient to compute the distribution (e.g. standard deviation not reported)
ND: % of severe patients not determined due to lack of information regarding the % of not early abstainers and/or the % of H/VH DRL patients
^1^: % determined based on reported mean, standard deviation and assuming a normal distribution

## Table S5: Reason for inclusion/exclusion

| **Study** | **Study title** | **Inclusion/Reason for exclusion** |
| --- | --- | --- |
| Adamson et al., 2015 | A randomized trial of combined citalopram and naltrexone for nonabstinent outpatients with co-occurring alcohol dependence and major depression | Patients included had psychiatric disorders, poly-addiction or hepatic dysfunctions |
| Addolorato et al. , 1999 | Gamma-hydroxybutyric acid (GHB) in the treatment of alcohol withdrawal syndrome: a randomized comparative study versus benzodiazepine. | Maintenance of abstinence was not the treatment goal |
| Addolorato et al., 2002 | Baclofen efficacy in reducing alcohol craving and intake: a preliminary double-blind randomized controlled study | Included |
| Addolorato et al., 2007 | Effectiveness and safety of baclofen for maintenance of alcohol abstinence in alcohol-dependent patients with liver cirrhosis: randomised, double-blind controlled study | Patients included had psychiatric disorders, poly-addiction or hepatic dysfunctions |
| Addolorato et al., 2011 | Dose–response effect of baclofen in reducing daily alcohol intake in alcohol dependence: secondary analysis of a randomized, double-blind, placebo-controlled trial. | Meta-analysis / Reanalysis of already screened studies |
| Ahmadi et al., 2004 | Naltrexone for alcohol-dependent patients. | Maintenance of abstinence was not the treatment goal |
| Ahmadi et al., 2009 | Predictors of treatment outcome in outpatient cocaine and alcohol dependence treatment. | Not conducted in the treatment of AD only |
| Ahmadi and Ahmadi, 2002 | A Double Blind, Placebo-Controlled Study of Naltrexone in the Treatment of Alcohol Dependence | Abstinence rate not available or available with a different definition |
| Annemans et al., 2000 | Economic evaluation of Campral (Acamprosate) compared to placebo in maintaining abstinence in alcohol-dependent patients | Meta-analysis / Reanalysis of already screened studies |
| Anthenelli et al., 2017 | A Randomized Trial Evaluating Whether Topiramate Aids Smoking Cessation and Prevents Alcohol Relapse in Recovering Alcohol-Dependent Men | Maintenance of abstinence was not the main treatment goal |
| Anton et al., 1996 | The obsessive compulsive drinking scale: A new method of assessing outcome in alcoholism treatment studies. | Abstinence rate not available or available with a different definition |
| Anton et al., 1999 | Naltrexone and cognitive behavioral therapy for the treatment of outpatient alcoholics: results of a placebo-controlled trial | Included |
| Anton et al., 2001 | Post treatment Results of Combining Naltrexone with Cognitive-Behavior Therapy for the Treatment of Alcoholism | Maintenance of abstinence was not the treatment goal |
| Anton et al., 2004 | A Multi-site Dose Ranging Study of Nalmefene in the Treatment of Alcohol Dependence | Maintenance of abstinence was not the treatment goal Abstinence rate not available or available with a different definition |
| Anton et al., 2005 | Naltrexone combined with either cognitive behavioral or motivational enhancement therapy for alcohol dependence. | Abstinence rate not available or available with a different definition |
| Anton et al., 2006 | Combined pharmacotherapies and behavioral interventions for alcohol dependence: The COMBINE study: A randomized controlled trial. | Meta-analysis / Reanalysis of already screened studies |
| Anton et al.,2008 | An evaluation of mu-opioid receptor (OPRM1) as a predictor of naltrexone response in the treatment of alcohol dependence: results from the Combined Pharmacotherapies and Behavioral Interventions for Alcohol Dependence (COMBINE) study | Meta-analysis / Reanalysis of already screened studies |
| Anton et al., 2008 | A Randomized, Multicenter, Double-Blind, Placebo-Controlled Study of the Efficacy and Safety of Aripiprazole for the Treatment of Alcohol Dependence | Included |
| Anton et al., 2009 | Efficacy of a combination of flumazenil and gabapentin in the treatment of alcohol dependence: relationship to alcohol withdrawal symptoms | Route of administration different than oral |
| Anton et al.,2011 | Gabapentin Combined With Naltrexone for the Treatment of Alcohol Dependence | Abstinence rate not available or available with a different definition |
| Anton et al., 2017 | Aripiprazole Suppression of Drinking in a Clinical Laboratory Paradigm: Influence of Impulsivity and Self-Control | Maintenance of abstinence was not the treatment goal |
| Arbaizar et al., 2010 | Topiramate in the treatment of alcohol dependence: a meta-analysis | Meta-analysis / Reanalysis of already screened studies |
| Arias et al., 2010 | Placebo-controlled trial of zonisamide for the treatment of alcohol dependence. | Abstinence rate not available or available with a different definition |
| Arias et al., 2014 | Pharmacogenetics of naltrexone and disulfiram in alcohol dependent, dually diagnosed veterans. | Patients included had psychiatric disorders, poly-addiction or hepatic dysfunctions Maintenance of abstinence was not the treatment goal |
| Ashare et al., 2019 | Placebo-controlled randomized clinical trial testing the efficacy and safety of varenicline for smokers with HIV | Not conducted in the treatment of AD |
| Atkinson et al., 2013 | Effects of disulfiram on QTc interval in non-opioid-dependent and methadone-treated cocaine-dependent patients. | Not conducted in the treatment of AD |
| Azorin et al., 2010 | Possible new ways in the pharmacological treatment of bipolar disorder and comorbid alcoholism | Patients included had psychiatric disorders, poly-addiction or hepatic dysfunctions |
| Balldin et al., 2003 | A 6-Month Controlled Naltrexone Study: Combined Effect With Cognitive Behavioral Therapy in Outpatient Treatment of Alcohol Dependence | Maintenance of abstinence was not the treatment goal |
| Baltieri et al., 2003 | Acamprosate in Alcohol Dependence:A Randomized Controlled Efficacy Study in a Standard Clinical Setting | Included |
| Baltieri & de Andrade, 2003 | Efficacy of acamprosate in the treatment of alcohol-dependent outpatients | Included |
| Baltieri et al., 2008 | Comparing topiramate with naltrexone in the treatment of alcohol dependence | Included |
| Barrias et al., 1997 | Acamprosate: multicenter Portuguese efficacy and tolerance evaluation study | Publication not in English |
| Baros et al., 2008 | Naltrexone and cognitive behavioral therapy for the treatment of alcohol dependence: do sex differences exist? | Meta-analysis / Reanalysis of already screened studies |
| Beck 2018 | Effects of high-dose baclofen on cue reactivity in alcohol dependence: A randomized, placebo-controlled pharmaco-fMRI study | Maintenance of abstinence was not the treatment goal |
| Bender et al., 2007 | The efficacy of the dopamine D2/D3 antagonist tiapride in maintaining abstinence: a randomized, double-blind, placebo-controlled trial in 299 alcohol-dependent patients | Included |
| Beraha et al., 2016 | Efficacy and safety of high-dose baclofen for the treatment of alcohol dependence: A multicentre, randomised, double-blind controlled trial | Maintenance of abstinence was not the treatment goal |
| Beraha et al., 2018 | Can baclofen change alcohol-related cognitive biases and what is the role of anxiety herein? | Not conducted in the treatment of AD |
| Berger et al., 2013 | Efficacy of Acamprosate for Alcohol Dependence in a Family Medicine Setting in the United States: A Randomized, Double-Blind, Placebo-Controlled Study | Maintenance of abstinence was not the treatment goal Abstinence rate not available or available with a different definition |
| Berger et al., 2016 | Alcohol Use Disorder Treatment: The Association of Pretreatment Use and the Role of Drinking Goal | Meta-analysis / Reanalysis of already screened studies |
| Besson,1994 | Combined efficacy of acamprosate and disulfiram for enhancing abstinence of chronic alcoholic patients during a one year post detoxification period | Study not found |
| Besson et al., 1998 | Combined Efficacy of Acamprosate and Disulfiram inthe Treatment of Alcoholism: A Controlled Study | Included |
| Bidwell et al., 2017 | ADHD symptoms impact smoking outcomes and withdrawal in response to Varenicline treatment for smoking cessation* | Not conducted in the treatment of AD |
| Bisaga et al., 2006 | A randomized placebo-controlled trial of gabapentin for cocaine dependence. | Not conducted in the treatment of AD |
| Bisaga et al., 2018 | Outpatient transition to extended-release injectable naltrexone for patients with opioid use disorder: A phase 3 randomized trial | Not conducted in the treatment of AD |
| Boeijinga et al., 2004 | Pharmacodynamic effects of acamprosate on markers of cerebral function in alcohol-dependent subjects administered as pretreatment and during alcohol abstinence. | Not conducted in the treatment of AD/ Maintenance of abstinence was not the treatment goal |
| Boothby et al., 2005 | Acamprosate for the treatment of alcohol dependence | Meta-analysis / Reanalysis of already screened studies |
| Borg, 1994 | Study title not available | Unpublished data |
| Borg, 2003 | Randomized controlled trial of acamprosate versus placebo in Swedish alcoholics [unpublished] | Unpublished data |
| Brady et al., 2002 | The use of divalproex in alcohol relapse prevention: a pilot study | Included |
| Brady et al., 2005 | Sertraline in the treatment of co-occurring alcohol dependence and posttraumatic stress disorder. | Patients included had psychiatric disorders, poly-addiction or hepatic dysfunctions |
| Brambilla et al., 2012 | [Gamma-hydroxybutyrate (GHB) for mid/long term treatment of alcohol dependence: a systematic review]. | Publication not in English |
| Brasser et al., 2004 | Alcohol effects during acamprosate treatment: a dose-response study in humans | Not double blind, randomized, placebo-controlled study |
| Brennan et al., 2013 | Clinical effectiveness of baclofen for the treatment of alcohol dependence: a review | Meta-analysis / Reanalysis of already screened studies |
| Brink et al., 2014 | Long-term efficacy, tolerability and safety of nalmefene as needed in patients with alcohol dependence: a 1-year, randomised controlled study. | Maintenance of abstinence was not the treatment goal |
| Briones et al., 2018 | Varenicline treatment for methamphetamine dependence: A randomized, double-blind phase II clinical trial | Not conducted in the treatment of AD |
| Brower et al., 2008 | A Randomized Double-Blind Pilot Trial of Gabapentin vs. Placebo to Treat Alcohol Dependence and Comorbid Insomnia | Patients included had psychiatric disorders, poly-addiction or hepatic dysfunctions |
| Bschor et al., 2018 | Baclofen for alcohol use disorder—a systematic meta-analysis | Meta-analysis / Reanalysis of already screened studies |
| Budzyński et al., 2000 | Naltrexone exerts a favourable effect on plasma lipids in abstinent patients with alcohol dependence | Not conducted in the treatment of AD/ Maintenance of abstinence was not the treatment goal |
| Caputo et al., 2003 | Gamma-hydroxybutyric acid versus naltrexone in maintaining alcohol abstinence: an open randomized comparative study. | Not double blind, randomized, placebo-controlled study |
| Caputo et al., 2007 | Comparing and combining gammahydroxybutyric acid (GHB) and naltrexone in maintaining abstinence from alcohol: an open randomised comparative study. | Not double blind, randomized, placebo-controlled study |
| Carroll et al., 1993 | Pharmacologic intervention for alcohol- and cocaine-abusing individuals: a pilot study of disulfiram vs. naltrexone. | Patients included had psychiatric disorders, poly-addiction or hepatic dysfunctions |
| Carroll 2016 | A randomized factorial trial of disulfiram and contingency management to enhance cognitive behavioral therapy for cocaine dependence. | Not conducted in the treatment of AD |
| Ceccanti et al., 1996 | Efficacy of therapeutic protocols for the treatment of alcohol withdrawal syndrome (AWS) | Maintenance of abstinence was not the treatment goal |
| Charney et al., 1984 | Naltrexone precipitated opiate withdrawal in methadone addicted human subjects: evidence for noradrenergic hyperactivity | Not conducted in the treatment of AD |
| Cheskin et al., 1995 | Assessment of nalmefene glucuronide as a selective gut opioid antagonist | Not conducted in the treatment of AD |
| Chick et al., 1992 | Disulfiram treatment of alcoholism. | Not double blind, randomized, placebo-controlled study |
| Chick et al., 2000 | A multicentre, randomized, double-blind, placebo-controlled trial of naltrexone in the treatment of alcohol dependence or abuse. | Included |
| Chick et al., 2000 | United Kingdom Multicentre Acamprosate Study (UKMAS): a 6-month prospective study of acamprosate versus placebo in preventing relapse after withdrawal from alcohol | Included |
| Childs et al., 2012 | Varenicline potentiates alcohol-induced negative subjective responses and offsets impaired eye movements | Not conducted in the treatment of AD |
| CHP et al., 2002 | Trial CPH-101-0701 | Maintenance of abstinence was not the treatment goal |
| CHP et al., 2004 | Trail CPH-101-0399 | Maintenance of abstinence was not the treatment goal |
| Clark et al., 1989 | Does lithium carbonate therapy for alcoholism deter relapse drinking? | Patients included had psychiatric disorders, poly-addiction or hepatic dysfunctions |
| Collins et al., 2014 | Harm reduction with pharmacotherapy for homeless people with alcohol dependence: protocol for a randomized controlled trial. | Not double blind, randomized, placebo-controlled study Route of administration different than oral Maintenance of abstinence was not the treatment goal |
| Colombo et al., 2004 | Role of GABA(B) receptor in alcohol dependence: reducing effect of baclofen on alcohol intake and alcohol motivational properties in rats and amelioration of alcohol withdrawal syndrome and alcohol craving in human alcoholics. | Maintenance of abstinence was not the treatment goal |
| Connery 2015 | Medication-assisted treatment of opioid use disorder: review of the evidence and future directions. | Not conducted in the treatment of AD |
| Cornelius et al., 1997 | Fluoxetine in depressed alcoholics: a double-blind, placebo controlled trial. | Patients included had psychiatric disorders, poly-addiction or hepatic dysfunctions |
| Corrêa et al., 2013 | A pilot study of full-dose ondansetron to treat heavy-drinking men withdrawing from alcohol in Brazil | Abstinence rate not available or available with a different definition |
| Crist et al., 2016 | A delta-opioid receptor genetic variant is associated with abstinence prior to and during cocaine dependence treatment. | Not conducted in the treatment of AD |
| Croissant et al., 2006 | A pilot study of oxcarbazepine versus acamprosate in alcohol-dependent patients | Not double blind, randomized, placebo-controlled study |
| Davidson et al., 2004 | Naltrexone and brief counseling to reduce heavy drinking in hazardous drinkers. | Maintenance of abstinence was not the treatment goal |
| Davidson et al., 2007 | Naltrexone's suppressant effects on drinking are limited to the first 3 months of treatment | Not double blind, randomized, placebo-controlled study |
| Dawes et al., 2005 | A prospective, open-label trial of ondansetron in adolescents with alcohol dependence | Not double blind, randomized, placebo-controlled study |
| de Goes e Castro, 2004 | Randomized, double-blind clinical trial with naltrexone and brief therapy for the in-patient of alcohol dependence [Portuguese Article] | Publication not in English |
| de la Fuente, 1989 | Acontrolled study of lithium carbonate in the treatment of alcoholism. | Patients included had psychiatric disorders, poly-addiction or hepatic dysfunctions |
| De Martini et al., 2014 | Predictors of pretreatment commitment to abstinence: results from the COMBINE study | Meta-analysis / Reanalysis of already screened studies |
| De Sousa et al., 2004 | A one-year pragmatic trial of naltrexone vs disulfiram in the treatment of alcohol dependence. | Not double blind, randomized, placebo-controlled study |
| De Sousa et al., 2005 | An open randomized study comparing disulfiram and acamprosate in the treatment of alcohol dependence. | Not double blind, randomized, placebo-controlled study |
| De Sousa et al., 2008 | An open randomized trial comparing disulfiram and naltrexone in adolescents with alcohol dependence. | Not double blind, randomized, placebo-controlled study |
| De Sousa et al., 2008 | An open randomized trial comparing disulfiram and topiramate in the treatment of alcohol dependence. | Not double blind, randomized, placebo-controlled study |
| De Wildt et al., 2002 | Does psychosocial treatment enhance the efficacy of acamprosate in patients with alcohol problems? | Not double blind, randomized, placebo-controlled study |
| Di bello 1995 | Gamma-hydroxybutyric acid induced suppression and prevention of alcohol withdrawal syndrome and relief of craving in alcohol dependent patients | Included |
| Dogrell et al., 2006 | Which treatment for alcohol dependence: naltrexone, acamprosate and/or behavioural intervention? | Meta-analysis / Reanalysis of already screened studies |
| Donovan et al., 2008 | Combined pharmacotherapies and behavioral interventions for alcohol dependence (The COMBINE Study): examination of posttreatment drinking outcomes | Meta-analysis / Reanalysis of already screened studies |
| Dorus et al., 1989 | Lithium treatment of depressed and nondepressed alcoholics. | Patients included had psychiatric disorders, poly-addiction or hepatic dysfunctions |
| Dranitsaris et al., 2009 | Meta-Analyses of Placebo-Controlled Trials of Acamprosate for the Treatment of Alcohol Dependence impact of the combined pharmacotherapies and behavior interventions study | Meta-analysis / Reanalysis of already screened studies |
| Dundon et al., 2008 | The therapeutic alliance in medical-based interventions impacts outcome in treating alcohol dependence | Meta-analysis / Reanalysis of already screened studies |
| Durant et al., 2018 | Using Baclofen to Explore GABA-B Receptor Function in Alcohol Dependence: Insights From Pharmacokinetic and Pharmacodynamic Measures | Not conducted in the treatment of AD |
| Elkashef et al., 2012 | Topiramate for the treatment of methamphetamine addiction: a multi-center placebo-controlled trial. | Not conducted in the treatment of AD |
| Elsing et al., 1996 | Randomized controlled trial for the treatment of alcohol withdrawal syndrome: clomethiazole vs. gamma-hydroxybutyric acid. | Maintenance of abstinence was not the treatment goal |
| Ernst et al., 2008 | An Intervention for Treating Alcohol Dependence: Relating Elements of Medical Management to Patient Outcomes With Implications for Primary Care | Meta-analysis / Reanalysis of already screened studies |
| Erwin & Slaton, 2014 | Varenicline in the Treatment of Alcohol Use Disorders | Meta-analysis / Reanalysis of already screened studies |
| Falk et al., 2014 | Cumulative Proportion of Responders Analysis (CPRA) as a Tool to Assess Treatment Outcome in Alcohol Clinical Trials | Meta-analysis / Reanalysis of already screened studies |
| Falk et al., 2019 | Evaluation of Drinking Risk Levels as Outcomes in Alcohol Pharmacotherapy Trials: A Secondary Analysis of 3 Randomized Clinical Trials | Meta-analysis / Reanalysis of already screened studies |
| Falk et al., 2019 | Gabapentin Enacarbil Extended-Release for Alcohol Use Disorder: A Randomized, Double-Blind, Placebo-Controlled, Multisite Trial Assessing Efficacy and Safety | Maintenance of abstinence was not the treatment goal |
| Farren et al., 2002 | A pilot double blind placebo controlled trial of sertraline with naltrexone in the treatment of opiate dependence | Not conducted in the treatment of AD |
| Farren et al., 2009 | A double-blind, placebo-controlled study of sertraline with naltrexone for alcohol dependence | Not double blind, randomized, placebo-controlled study |
| Ferri et al., 1991 | Association between GHB and disulfiram for the treatment of alcohol dependence | Not double blind, randomized, placebo-controlled study |
| Fertig et al., 2012 | A Double-Blind, Placebo-Controlled Trial Assessing the Efficacy of Levetiracetam Extended-Release in Very Heavy Drinking Alcohol-Dependent Patients | Included |
| Flanerry et al., 2004 | Baclofen for alcohol dependence: a preliminary open-label study | Not double blind, randomized, placebo-controlled study |
| Florez et al., 2008 | Using topiramate or naltrexone for the treatment of alcohol-dependent patients. | Not double blind, randomized, placebo-controlled study |
| Florez et al., 2011 | Topiramate for the treatment of alcohol dependence: comparison with naltrexone. | Not double blind, randomized, placebo-controlled study |
| Foulds et al., 2015 | Depression outcome in alcohol dependent patients: an evaluation of the role of independent and substance-induced depression and other predictors | Not conducted in the treatment of AD |
| Foulds et al., 2015 | OPRM1 genotype and naltrexone response in depressed alcohol-dependent patients | Patients included had psychiatric disorders, poly-addiction or hepatic dysfunctions |
| Foulds et al., 2016 | Personality Predictors of Drinking Outcomes in Depressed Alcohol-Dependent Patients | Patients included had psychiatric disorders, poly-addiction or hepatic dysfunctions |
| Fox et al., 2012 | Prazosin effects on stress- and cue-induced craving and stress response in alcohol-dependent individuals: preliminary findings | Maintenance of abstinence was not the treatment goal |
| Fucito et al., 2011 | A preliminary investigation of varenicline for heavy drinking smokers | Publication not in English |
| Fuller et al., 1979 | Disulfiram for the treatment of alcoholism. An evaluation in 128 men. | Not double blind, randomized, placebo-controlled study |
| Fuller et al., 1986 | Disulfiram treatment of alcoholism. A Veterans Administration cooperative study. | Not double blind, randomized, placebo-controlled study |
| Furieri et al., 2007 | Gabapentin reduces alcohol consumption and craving: a randomized, double-blind, placebo-controlled trial. | Maintenance of abstinence was not the treatment goal |
| Galarza et al., 1997 | The use of natlrexone to treat ambulatory patients with alcohol dependence | Abstinence rate not available or available with a different definition |
| Gallimberti et al.,1989 | Gamma-hydroxybutyric acid for treatment of alcohol withdrawal syndrome | Maintenance of abstinence was not the treatment goal |
| Gallimberti et al.,1992 | Gamma-Hydroxybutyric Acid in the Treatment of Alcohol Dependence: A Double-Blind Study | Not clear if the maintenance of abstinence was not the treatment goal |
| Gandhi et al., 2020 | The Impact of Varenicline on Alcohol Consumption in Subjects With Alcohol Use Disorders: Systematic Review and Meta-Analyses | Meta-analysis / Reanalysis of already screened studies |
| Garbutt et al., 2005 | Efficacy and Tolerability of Long-Acting Injectable Naltrexone for Alcohol Dependence A Randomized Controlled Trial | Maintenance of abstinence was not the treatment goal Abstinence rate not available or available with a different definition |
| Garbutt et al., 1999 | Pharmacological Treatment of Alcohol Dependence A Review of the Evidence | Meta-analysis / Reanalysis of already screened studies |
| Garbutt et al., 2007 | Placebo-controlled trial of baclofen in alcohol dependence | Conference paper |
| Garbutt et al., 2010 | A double-blind, randomized, placebo-controlled pilot study of baclofen combined with naltrexone for alcohol dependence | Study not found |
| Garbutt et al., 2010 | Efficacy and Safety of Baclofen for Alcohol Dependence: A Randomized, Double-Blind, Placebo-Controlled Trial | Maintenance of abstinence was not the treatment goal Abstinence rate not available or available with a different definition |
| Garbutt et al., 2016 | Association of the Sweet-Liking Phenotype and Craving for Alcohol With the Response to Naltrexone Treatment in Alcohol Dependence A Randomized Clinical Trial | Route of administration different than oral |
| Gastpar et al., 2002 | Lack of Efficacy of Naltrexone in the Prevention of Alcohol Relapse: Results From a German Multicenter Study | Included |
| Geerlings et al., 1997 | Acamprosate and Prevention of Relapse in Alcoholics Results of a Randomized, Placebo-Controlled, Double-Blind Study in Out-Patient Alcoholics in the Netherlands, Belgium and Luxembourg | Included |
| Geisel et al., 2019 | Total and acylated ghrelin plasma levels as potential long-term response markers in alcohol-dependent patients receiving high-dose of the GABA-B receptor agonist baclofen | Meta-analysis / Reanalysis of already screened studies |
| George et al., 2000 | Predictability of alcohol relapse by hippocampal volumetry and psychometric variables. | Not conducted in the treatment of AD |
| George et al., 2000 | Disulfiram versus placebo for cocaine dependence in buprenorphine-maintained subjects: a preliminary trial | Not conducted in the treatment of AD |
| Gerra et al., 1992 | Effects of fluoxetine and caacetyl-homotaurinate on alcohol intake in familial and nonfamilial alcoholic patients. | Not double blind, randomized, placebo-controlled study |
| Gonzàles et al., 2007 | Clinical efficacy of gabapentin versus tiagabine for reducing cocaine use among cocaine dependent methadone-treated patients | Not conducted in the treatment of AD |
| Gorelick et al., 1992 | Effect of fluoxetine on alcohol consumption in male alcoholics. | Not double blind, randomized, placebo-controlled study |
| Grant et al., 2017 | Naltrexone and Disulfiram Treatment Response in Veterans With Alcohol Dependence and Co-Occurring Problem-Gambling Features | Patients included had psychiatric disorders, poly-addiction or hepatic dysfunctions |
| Green & Ray, 2018 | Effects of varenicline on subjective craving and relative reinforcing value of cigarettes | Not conducted in the treatment of AD |
| Greenfield et al., 2010 | Gender differences in alcohol treatment: an analysis of outcome from the COMBINE study | Meta-analysis / Reanalysis of already screened studies |
| Gross et al., 2013 | Predictability of alcohol relapse by hippocampal volumetry and psychometric variables. | Not conducted in the treatment of AD |
| Gual et al., 2013 | A randomised, double-blind, placebo-controlled, efficacy study of nalmefene, as-needed use, in patients with alcohol dependence. | Maintenance of abstinence was not the treatment goal |
| Gual & Lehert, 2001 | Acamprosate during and after acute alcohol withdrawal: a double-blind placebo-controlled study in Spain | Patients were not detoxified/abstinent at treatment initiation |
| Guardia et al., 2002 | A Double-Blind, Placebo-Controlled Study of Naltrexone in the Treatment of Alcohol-Dependence Disorder: Results from a Multicenter Clinical Trial | Abstinence rate not available or available with a different definition |
| Guardia et al., 2004 | Double-Blind, Placebo-Controlled Study of Olanzapine in the Treatment of Alcohol-Dependence Disorder | Maintenance of abstinence was not the treatment goal Abstinence rate not available or available with a different definition |
| Guardia et al., 2011 | A double-blind, placebo-controlled, randomized pilot study comparing quetiapine with placebo, associated to naltrexone, in the treatment of alcohol-dependent patients | Not double blind, randomized, placebo-controlled study (no arm treated only with placebo) |
| Gueorguieva et al., 2011 - Mild | Baseline trajectories of drinking moderate acamprosate and naltrexone effects in the COMBINE study. | Included |
| Gueorguieva et al., 2011 - Severe | Baseline trajectories of drinking moderate acamprosate and naltrexone effects in the COMBINE study. | Included |
| Gueorguieva et al., 2015 | An analysis of moderators in the COMBINE study: Identifying subgroups of patients who benefit from acamprosate | Meta-analysis / Reanalysis of already screened studies |
| Hämmerberg et al., 2004 | A comparison of two intensities of psychosocial intervention for alcohol dependent patients treated with acamprosate. | Not double blind, randomized, placebo-controlled study |
| Hammond et al., 2015 | Anticonvulsants for the Treatment of Alcohol Withdrawal Syndrome and Alcohol Use Disorders | Meta-analysis / Reanalysis of already screened studies |
| Hartzleret al., 2011 | Self-Efficacy Change as a Mediator of Associations Between Therapeutic Bond and One-Year Outcomes in Treatments for Alcohol Dependence | Meta-analysis / Reanalysis of already screened studies |
| Haung, 2002 | Placebo-controlled trial of naltrexone in outpatient treatment of alcohol dependence | Study not found |
| Hauser et al., 2017 | The Safety and Efficacy of Baclofen to Reduce Alcohol Use in Veterans with Chronic Hepatitis C: A Randomized Clinical Trial | Not conducted in the treatment of AD Patients included had psychiatric disorders, poly-addiction or hepatic dysfunctions |
| Hays et al., 2011 | Varenicline for tobacco dependence treatment in recovering alcohol-dependent smokers: An open-label pilot study | Not double blind, randomized, placebo-controlled study Not conducted in the treatment of AD |
| Heinala et al., 2001 | Targeted Use of Naltrexone Without Prior Detoxification in the Treatment of Alcohol Dependence: A Factorial Double-Blind, Placebo-Controlled Trial | Abstinence rate not available or available with a different definition |
| Hermann et al., 2017 | Low μ-Opioid Receptor Status in Alcohol Dependence Identified by Combined Positron Emission Tomography and Post-Mortem Brain Analysis | Not conducted in the treatment of AD |
| Hersh et al., 1998 | Naltrexone treatment of comorbid alcohol and cocaine use disorders | Patients included had psychiatric disorders, poly-addiction or hepatic dysfunctions |
| Higuchi, 2015 | Naltrexone treatment of comorbid alcohol and cocaine use disorders | Patients included had psychiatric disorders, poly-addiction or hepatic dysfunctions |
| Higuchi, 2015 | Efficacy of acamprosate for the treatment of alcohol dependence long after recovery from withdrawal syndrome: a randomized, double-blind, placebo-controlled study conducted in Japan (Sunrise Study) | Included |
| Huang et al., 2005 | A double-blind, placebo-controlled study of naltrexone in the treatment of alcohol dependence in Taiwan | Abstinence rate not available or available with a different definition |
| Hurt et al., 2018 | Varenicline for tobacco-dependence treatment in alcohol-dependent smokers: A randomized controlled trial | Not conducted in the treatment of AD |
| Hutchison et al., 2006 | The effect of olanzapine on craving and alcohol consumption | Maintenance of abstinence was not the treatment goal |
| Indave et al., 2016 | Antipsychotic medications for cocaine dependence (Review) | Not conducted in the treatment of AD |
| Isgro et al., 2017 | Type A/Type B Alcoholism Predicts Differential Response to Topiramate in a Smoking Cessation Trial in Dually Diagnosed Men | Not conducted in the treatment of AD |
| Janiri et al., 1996 | Effects of fluoxetine at antidepressant doses on short-term outcome of detoxified alcoholics. | Patients included had psychiatric disorders, poly-addiction or hepatic dysfunctions |
| Jao et al., 2017 | Does menthol cigarette use moderate the effect of nicotine metabolism on short-term smoking cessation? | Not conducted in the treatment of AD |
| Jarosz et al., 2013 | Naltrexone (50 mg) plus psychotherapy in alcohol-dependent patients: a meta-analysis of randomized controlled trials | Meta-analysis / Reanalysis of already screened studies |
| Jaury et al., 2017 | Le baclofène est-il efficace dans le traitement de l’alcoolisme ? L’étude Bacloville | Publication not in English |
| Johnsen et al., 1987 | A double-blind placebo controlled study of male alcoholics given a subcutaneous disulfiram implantation. | Route of administration different than oral |
| Johnsen et al., 1991 | Disulfiram implant: A double-blind placebo controlled follow-up on treatment outcome. | Route of administration different than oral |
| Johnson et al., 2000 | Ondansetron for reduction of drinking among biologically predisposed alcoholic patients: A randomized controlled trial. | Maintenance of abstinence was not the treatment goal |
| Johnson et al., 2004 | Oral Topiramate Reduces the Consequences of Drinking and Improves the Quality of Life of Alcohol-Dependent Individuals A Randomized Controlled Trial | Abstinence rate not available or available with a different definition |
| Johnson et al., 2002 | Ondansetron reduces the craving of biologically predisposed alcoholics | Meta-analysis / Reanalysis of already screened studies |
| Johnson et al., 2003 | Ondansetron reduces mood disturbance among biologically predisposed, alcohol-dependent individuals | Abstinence rate not available or available with a different definition |
| Johnson et al., 2003 | Oral topiramate for treatment of alcohol dependence: a randomised controlled trial | Maintenance of abstinence was not the treatment goal |
| Johnson et al., 2005 | Use of oral topiramate to promote smoking abstinence among alcohol-dependent smokers: a randomized controlled trial. | Not conducted in the treatment of AD |
| Johnson et al., 2006 | A preliminary randomized, double-blind, placebo-controlled study of the safety and efficacy of ondansetron in the treatment of cocaine dependence | Not conducted in the treatment of AD |
| Johnson et al., 2007 | Topiramate for Treating Alcohol Dependence A Randomized Controlled Trial | Maintenance of abstinence was not the treatment goal |
| Johnson et al., 2013 | Determination of genotype combinations that can predict the outcome of the treatment of alcohol dependence using the 5-HT(3) antagonist ondansetron | Meta-analysis / Reanalysis of already screened studies |
| Jørgensen et al., 2011 | The efficacy of disulfiram for the treatment of alcohol use disorder | Meta-analysis / Reanalysis of already screened studies |
| Kabel et al., 1996 | placebo-controlled, double blind study of fluoxetine in severe alcohol dependence | Abstinence rate not available or available with a different definition |
| Kahler et al., 2017 | A Double-Blind Randomized Placebo-Controlled Trial of Oral Naltrexone for Heavy-Drinking Smokers Seeking Smoking Cessation Treatment. | Maintenance of abstinence was not the treatment goal |
| Kahn et al., 2009 | Multi-center trial of baclofen for abstinence initiation in severe cocaine-dependent individuals. | Not conducted in the treatment of AD |
| Kampman et al., 2013 | A double-blind, placebo-controlled trial of topiramate for the treatment of comorbid cocaine and alcohol dependence. | Patients included had psychiatric disorders, poly-addiction or hepatic dysfunctions Maintenance of abstinence was not the treatment goal |
| Kampman et al., 2004 | A pilot trial of topiramate for the treatment of cocaine dependence. | Not conducted in the treatment of AD |
| Kampman et al., 2007 | A Double-Blind, Placebo-Controlled Pilot Trial of Quetiapine for the Treatment of Type A and Type B Alcoholism | Included |
| Kampman et al., 2011 | A double-blind, placebo-controlled pilot trial of acamprosate for the treatment of cocaine dependence. | Not conducted in the treatment of AD |
| Karhuvaara et al., 2007 | Targeted nalmefene with simple medical management in the treatment of heavy drinkers: A randomized double-blind placebo-controlled multicenter study. | Maintenance of abstinence was not the treatment goal |
| Khoramizadeh et al., 2019 | Treatment of amphetamine abuse/use disorder: a systematic review of a recent health concern | Not conducted in the treatment of AD |
| Kiefer et al., 2003 | A Double-Blind, Placebo-Controlled Pilot Trial of Quetiapine for the Treatment of Type A and Type B Alcoholism | Included |
| Kiefer et al., 2004 | Long-term effects of pharmacotherapy on relapse prevention in alcohol dependence | Included |
| Killeen et al., 2004 | Effectiveness of Naltrexone in a Community Treatment Program | Included |
| Kiritze-Topor et al., 2004 | A pragmatic trial of acamprosate in the treatment of alcohol dependence in primary care. | Not double blind, randomized, placebo-controlled study |
| Kishi et al., 2013 | Antipsychotics for primary alcohol dependence: a systematic review and meta-analysis of placebo-controlled trials. | Meta-analysis / Reanalysis of already screened studies |
| Klemperer et al., 2018 | Study characteristics influence the efficacy of substance abuse treatments: A meta-analysis of medications for alcohol use disorder | Meta-analysis / Reanalysis of already screened studies |
| Knapp et al., 2015 | Zonisamide, topiramate, and levetiracetam efficacy and neuropsychological effects in alcohol use disorders. | Maintenance of abstinence was not the treatment goal |
| Knox et al., 1999 | Using naltrexone in inpatient alcoholism treatment. | Abstinence rate not available or available with a different definition |
| Koeter et al., 2010 | Effect of early and late compliance on the effectiveness of acamprosate in the treatment of alcohol dependence | Abstinence rate not available or available with a different definition |
| Kranzler et al., 1994 | Buspirone treatment of anxious alcoholics. | Patients included had psychiatric disorders, poly-addiction or hepatic dysfunctions |
| Kranzler et al., 1995 | Placebo-controlled trial of fluoxetine as an adjunct to relapse prevention in alcoholics | Patients included had psychiatric disorders, poly-addiction or hepatic dysfunctions |
| Kranzler et al., 1998 | Sustained-Release Naltrexone for Alcoholism Treatment: A Preliminary Study | Maintenance of abstinence was not the treatment goal |
| Kranzler et al., 2000 | Naltrexone vs. Nefazodone for Treatment of Alcohol Dependence A Placebo-Controlled Trial | Included |
| Kranzler et al., 2003 | Targeted Naltrexone for Early Problem Drinkers | Maintenance of abstinence was not the treatment goal |
| Kranzler et al., 2004 | Naltrexone Depot for Treatment of Alcohol Dependence: A Multicenter, Randomized, Placebo-Controlled Clinical Trial | Route of administration different than oral |
| Kranzler et al., 2006 | Sertraline treatment of co-occurring alcohol dependence and major depression | Patients included had psychiatric disorders, poly-addiction or hepatic dysfunctions |
| Kranzler et al., 2009 | Targeted naltrexone for problem drinkers. | Maintenance of abstinence was not the treatment goal |
| Kranzler et al., 2014 | Topiramate treatment for heavy drinkers: moderation by a GRIK1 polymorphism | Maintenance of abstinence was not the treatment goal |
| Kranzler & Gage, 2008 | Acamprosate Efficacy in Alcohol-Dependent Patients: Summary of Results from Three Pivotal Trials | Meta-analysis / Reanalysis of already screened studies |
| Krupitsky et al., 2007 | Antiglutamatergic Strategies for Ethanol Detoxification: Comparison With Placebo and Diazepam | Not double blind, randomized, placebo-controlled study |
| Krupitsky et al., 2015 | Double blind placebo controlled randomized pilot clinical trial of baclofen (Baclosan(R)) for alcohol dependence. | Study not in English |
| Krupitsky et al., 2017 | Efficacy and Safety of the Use of Baclofen in the Treatment of Alcohol Dependent (a double-blind, randomized, placebo-controlled pilot study) | Maintenance of abstinence was not the treatment goal |
| Krystal et al., 2001 | Naltrexone in the treatment of alcohol dependence | Abstinence rate not available or available with a different definition |
| Laaksonen et al., 2008 | A randomized, multicentre, open-label, comparative trial of disulfiram, naltrexone and acamprosate in the treatment of alcohol dependence. | Not double blind, randomized, placebo-controlled study |
| Ladewig et al., 1993 | Acamprosate - a stabilising factor in the long-term treatment of alcoholics [in German] | Publication not in English |
| Landabaso et al., 1999 | Naltrexone in the treatment of alcoholism: two-year follow up results. | Abstinence rate not available or available with a different definition |
| Lapham et al., 2009 | The effects of extended-release naltrexone on holiday drinking in alcohol-dependent patients | Meta-analysis / Reanalysis of already screened studies |
| Latt et al., 2002 | Naltrexone in alcohol dependence: a randomised controlled trial of effectiveness in a standard clinical setting | Abstinence rate not available or available with a different definition |
| Lee et al., 2001 | Naltrexone in the treatment of male alcoholics– an effectiveness study in Singapore | Abstinence rate not available or available with a different definition |
| Leggio et al., 2010 | Effectiveness and Safety of Baclofen in the Treatment of Alcohol Dependent Patients | Meta-analysis / Reanalysis of already screened studies |
| Leggio et al., 2012 | Baclofen promotes alcohol abstinence in alcohol dependent cirrhotic patients with hepatitis C virus (HCV) infection | Meta-analysis / Reanalysis of already screened studies Patients included had psychiatric disorders, poly-addiction or hepatic dysfunctions |
| Leggio et al., 2015 | A preliminary double-blind, placebo-controlled randomized study of baclofen effects in alcoholic smokers | Maintenance of abstinence was not the treatment goal |
| Lejoyeux & Lehert, 2011 | Alcohol-Use Disorders and Depression: Results from Individual Patient Data Meta-Analysis of the Acamprosate-Controlled Studies | Meta-analysis / Reanalysis of already screened studies |
| Leone et al., 2010 | Gamma-hydroxybutyrate (GHB) for treatment of alcohol withdrawal and prevention of relapses (Review) | Meta-analysis / Reanalysis of already screened studies |
| Lesouef et al., 2014 | Efficacy of baclofen on abstinence and craving in alcohol-dependent patients: a meta-analysis of randomized controlled trials | Meta-analysis / Reanalysis of already screened studies |
| Levin et al., 2008 | Effects of major depressive disorder and attention-deficit/hyperactivity disorder on the outcome of treatment for cocaine dependence. | Not conducted in the treatment of AD |
| Levin et al., 2020 | Extended release mixed amphetamine salts and topiramate for cocaine dependence: A randomized clinical replication trial with frequent users | Not conducted in the treatment of AD |
| Lhuintre et al., 1985 | Ability of calcium bis acetyl homotaurine, a gaba agonist, to prevent relapse in weaned alcoholics | Abstinence rate not available or available with a different definition |
| Lhuintre et al., 1990 | Acamprosate appears to decrease alcohol intake in weaned alcoholics | Abstinence rate not available or available with a different definition |
| Likhitsathian et al., 2013 | Topiramate treatment for alcoholic outpatients recently receiving residential treatment programs: a 12-week, randomized, placebo-controlled trial. | Abstinence rate not available or available with a different definition |
| Litten et al., 2012 | A Double-Blind, Placebo-Controlled Trial to Assess the Efficacy of Quetiapine Fumarate in Very Heavy Drinking Alcohol-Dependent Patients | Maintenance of abstinence was not the treatment goal |
| Litten et al., 2013 | A Double-Blind, Placebo-Controlled Trial Assessing the Efficacy of Varenicline Tartrate for Alcohol Dependence | Maintenance of abstinence was not the treatment goal |
| Litten et al., 2013 | The Placebo Effect in Clinical Trials for Alcohol Dependence: An Exploratory Analysis of 51 Naltrexone and Acamprosate Studies | Meta-analysis / Reanalysis of already screened studies |
| Liu et al., 2009 | Subjective, cognitive/psychomotor, and physiological effects of aripiprazole in Chinese light and heavy smokers | Not conducted in the treatment of AD |
| Longabaugh et al., 2009 | Extended naltrexone and broad spectrum treatment or motivational enhancement therapy. | Not double blind, randomized, placebo-controlled study |
| Lukas et al., 2013 | Extended-release naltrexone (XR-NTX) attenuates brain responses to alcohol cues in alcohol-dependent volunteers: a bold FMRI study | Maintenance of abstinence was not the treatment goal |
| Ma et al., 2006 | Topiramate reduces the harm of excessive drinking: implications for public health and primary care. | Not conducted in the treatment of AD |
| Ma et al., 2013 | Fine-grain analysis of the treatment effect of topiramate on methamphetamine addiction with latent variable analysis | Not conducted in the treatment of AD |
| Malcolm et al., 1992 | A placebo controlled trial of buspirone in anxious inpatient alcoholics. | Patients included had psychiatric disorders, poly-addiction or hepatic dysfunctions |
| Malec et al., 1996 | Buspirone in the treatment of alcohol dependence. | Maintenance of abstinence was not the treatment goal |
| Mann et al., 2004 | The efficacy of acamprosate in the maintenance of abstinence in alcohol-dependent individuals: results of a meta-analysis | Meta-analysis / Reanalysis of already screened studies |
| Mann et al., 2012 | Results of a double-blind, placebo-controlled pharmacotherapy trial in alcoholism conducted in Germany and comparison with the US COMBINE study | Abstinence rate not available or available with a different definition |
| Mann et al., 2013 | A randomised, double-blind, placebo-controlled, efficacy study of nalmefene, as-needed use, in patients with alcohol dependence. | Maintenance of abstinence was not the treatment goal |
| Maremmani et al., 2011 | Long-Term γ-Hydroxybutyric Acid (GHB) and Disulfiram Combination Therapy in GHB Treatment-Resistant Chronic Alcoholics | Not double blind, randomized, placebo-controlled study |
| Mariani et al., 2016 | Pilot trial of gabapentin for the treatment of benzodiazepine abuse or dependence in methadone maintenance patients. | Not conducted in the treatment of AD |
| Marra et al., 2002 | Amisulpride Does Not Prevent Relapse in Primary Alcohol Dependence: Results of a Pilot Randomized, Placebo-Controlled Trial | Included |
| Martinotti et al., 2007 | High and low dosage oxcarbazepine versus naltrexone for the prevention of relapse in alcoholdependent patients. | Not double blind, randomized, placebo-controlled study |
| Martinotti et al., 2007 | Oxcarbazepine at high dosages for the treatment of alcohol dependence. | Not double blind, randomized, placebo-controlled study |
| Martinotti et al., 2009 | Aripiprazole in the treatment of patients with alcohol dependence: a double-blind, comparison trial vs. Naltrexone | Not double blind, randomized, placebo-controlled study |
| Martinotti et al., 2010 | Pregabalin versus naltrexone in alcohol dependence: a randomised, double-blind, comparison trial. | Not double blind, randomized, placebo-controlled study |
| Mason et al., 1994 | A Double-Blind, Placebo-Controlled Pilot Study to Evaluate the Efficacy and Safety of Oral Nalmefene HCl for Alcohol Dependence | Abstinence rate not available or available with a different definition |
| Mason et al., 1996 | A double-blind, placebo-controlled trial of desipramine for primary alcohol dependence stratified on the presence or absence of major depression. | Patients included had psychiatric disorders, poly-addiction or hepatic dysfunctions |
| Mason et al., 1999 | A double-blind, placebo-controlled study of oral nalmefene for alcohol dependence. | Abstinence rate not available or available with a different definition |
| Mason et al., 2000 | Acamprosate for the treatment of alcohol dependence: a review of double-blind, placebo-controlled trials | Meta-analysis / Reanalysis of already screened studies |
| Mason, 2001 | Treatment of alcohol-dependent outpatients with acamprosate: a clinical review. | Meta-analysis / Reanalysis of already screened studies |
| Mason, 2005 | Acamprosate in the treatment of alcohol dependence | Meta-analysis / Reanalysis of already screened studies |
| Mason et al., 2006 | Effect of oral acamprosate on abstinence in patients with alcohol dependence in a double-blind, placebo-controlled trial: The role of patient motivation | Maintenance of abstinence was not the treatment goal (“expressed a desire to cut down or stop drinking”) Abstinence rate not available or available with a different definition |
| Mason et al., 2009 | Proof-of-concept human laboratory study for protracted abstinence in alcohol dependence: effect of gabapentine | Not double blind, randomized, placebo-controlled study Abstinence rate not available or available with a different definition |
| Mason et al., 2012 | A proof-of-concept randomized controlled study of gabapentin: effects on cannabis use, withdrawal and executive function deficits in cannabis-dependent adults | Not conducted in the treatment of AD |
| Mason et al., 2014 | Gabapentin Treatment for Alcohol Dependence A Randomized Clinical Trial | Included |
| Mason et Crean, 2007 | Acamprosate in the treatment of alcohol dependence: clinical and economic considerations. | Meta-analysis / Reanalysis of already screened studies |
| Mason & Lehert, 2009 | Effects of Nicotine and Illicit Substance Use on Alcoholism - Treatment Outcomes and Acamprosate Efficacy | Not conducted in the treatment of AD Abstinence rate not available or available with a different definition |
| Mason & Lehert, 2012 | Acamprosate for Alcohol Dependence: A Sex-Specific Meta-Analysis Based on Individual Patient Data | Meta-analysis / Reanalysis of already screened studies |
| McKee et al., 2009 | Varenicline Reduces Alcohol Self-Administration in Heavy-Drinking Smokers | Not conducted in the treatment of AD |
| Mendelevich & Zalmunin, 2015 | Paradoxes of evidence in Russian addiction medicine | Not conducted in the treatment of AD |
| Merry et al., 1976 | Prophylactic treatment of alcoholism by lithium carbonate. | Not double blind, randomized, placebo-controlled study |
| Meszaros et al., 2013 | Varenicline Treatment of Concurrent Alcohol and Nicotine Dependence in Schizophrenia A Randomized, Placebo-Controlled Pilot Trial | Patients included had psychiatric disorders, poly-addiction or hepatic dysfunctions |
| Milivojevic et al., 2020 | Effects of Prazosin on Provoked Alcohol Craving and Autonomic and Neuroendocrine Response to Stress in Alcohol Use Disorder | Maintenance of abstinence was not the treatment goal |
| Miller et al., 2011 | Medical treatment of alcohol dependence: a systematic review | Meta-analysis / Reanalysis of already screened studies |
| Minozzi et al., 2006 | Oral naltrexone maintenance treatment for opioid dependence. | Abstinence rate not available or available with a different definition |
| Minozzi et al., 2018 | Baclofen for alcohol use disorders | Meta-analysis / Reanalysis of already screened studies |
| Mishra et al., 2010 | A study of comparative eKicacy of baclofen vs acamprosate in reducing alcohol craving and abuse. | Not double blind, randomized, placebo-controlled study |
| Mitchell et al., 2012 | Varenicline decreases alcohol consumption in heavy-drinking smokers | Not conducted in the treatment of AD Abstinence rate not available or available with a different definition |
| Moak et al., 2003 | Sertraline and cognitive behavioral therapy for depressed alcoholics: Results of a placebo controlled trial. | Patients included had psychiatric disorders, poly-addiction or hepatic dysfunctions |
| Monterosso et al., 2001 | Predicting Treatment Response to Naltrexone: The Influence of Craving and Family History | Abstinence rate not available or available with a different definition |
| Monti et al., 1999 | Naltrexone's effect on cue-elicited craving among alcoholics in treatment | Maintenance of abstinence was not the treatment goal |
| Monti et al., 2001 | Naltrexone and Cue Exposure With Coping and Communication Skills Training for Alcoholics: Treatment Process and 1-Year Outcomes | Maintenance of abstinence was not the treatment goal |
| Morgenstern et al., 2012 | A randomized clinical trial of naltrexone and behavioral therapy for problem drinking men who have sex with men. | Maintenance of abstinence was not the treatment goal |
| Morley et al., 2006 | Naltrexone versus acamprosate in the treatment of alcohol dependence: a multi-centre, randomized, double-blind, placebo-controlled trial | Included |
| Morley et al., 2009 | Sample bias from different recruitment strategies in a randomised controlled trial for alcohol dependence. | Not double blind, randomized, placebo-controlled study Not conducted in the treatment of AD |
| Morley et al., 2010 | Clinical Predictors of Outcome from an Australian Pharmacological Relapse Prevention Trial | Meta-analysis / Reanalysis of already screened studies |
| Morley et al., 2013 | The efficacy and biobehavioural basis of baclofen in the treatment of alcoholic liver disease (BacALD): study protocol for a randomised controlled trial. | Meta-analysis / Reanalysis of already screened studies |
| Morley et al., 2014 | Baclofen for the Treatment of Alcohol Dependence and Possible Role of Comorbid Anxiety | Maintenance of abstinence was not the treatment goal |
| Morley et al., 2018 | Moderation of baclofen response by a GABAB receptor polymorphism: Results from the BacALD randomized controlled trial | Meta-analysis / Reanalysis of already screened studies |
| Morley et al., 2018 | Baclofen in the treatment of alcohol dependence with or without liver disease: multisite, randomised, double-blind, placebo-controlled trial | Patients included had psychiatric disorders, poly-addiction or hepatic dysfunctions |
| Morley et al., 2018 | Neurometabolite Levels in Alcohol Use Disorder Patients During Baclofen Treatment and Prediction of Relapse to Heavy Drinking | Meta-analysis / Reanalysis of already screened studies |
| Morley et al., 2018 | Topiramate versus naltrexone for alcohol use disorder: study protocol for a genotype-stratified, double-blind randomised controlled trial (TOP study) | Not double blind, randomized, placebo-controlled study |
| Morris et al., 2001 | Naltrexone for alcohol dependence: a randomized controlled trial | Included |
| Mueller et al., 1997 | A Double-Blind, Placebo-Controlled Pilot Study of Carbamazepine for the Treatment of Alcohol Dependence | Abstinence rate not available or available with a different definition |
| Müller et al., 2015 | High-Dose Baclofen for the Treatment of Alcohol Dependence (BACLAD study): A Randomized, Placebo-Controlled Trial | Included |
| Murphy et al., 2017 | Effects of Varenicline versus Transdermal Nicotine Replacement Therapy on Cigarette Demand on Quit Day in Individuals with Substance Use Disorders | Not conducted in the treatment of AD |
| Muzyk et al., 2012 | Defining the Role of Baclofen for the Treatment of Alcohol Dependence A Systematic Review of the Evidence | Meta-analysis / Reanalysis of already screened studies |
| Myrick et al., 2010 | The effect of aripiprazole on cue-induced brain activation and drinking parameters in alcoholics | Maintenance of abstinence was not the treatment goal |
| Nalpas et al., 1990 | [Acamprosate. From pharmacology to therapeutics] | Meta-analysis / Reanalysis of already screened studies |
| Namkoong et al., 2003 | Acamprosate in Korean alcohol-dependent patients: a multi-centre, randomized, double-blind, placebo-controlled study. | Included |
| Naranjo et al., 1993 | Clinical pharmacology of serotonin-altering medications for decreasing alcohol consumption | Maintenance of abstinence was not the treatment goal |
| Naranjo et al., 1994 | Serotonin-altering medications and desire, consumption and effects of alcohol-treatment implications | Meta-analysis / Reanalysis of already screened studies |
| Narayana et al., 2008 | Use of Anti-Craving Agents in Soldiers with Alcohol Dependence Syndrome | Not double blind, randmized, placebo-controlled study |
| Nava et al., 2006 | Comparing treatments of alcoholism on craving and biochemical measures of alcohol consumptionst. | Not double blind, randomized, placebo-controlled study |
| Nava et al., 2007 | Gamma-hydroxybutyrate reduces both withdrawal syndrome and hypercortisolism in severe abstinent alcoholics: an open study vs diazepam. | Not double blind, randomized, placebo-controlled study |
| Ndegwa et al., 2016 | Injectable Extended-Release Naltrexone to Treat Opioid Use Disorder | Route of administration different than oral |
| Nestor et al, 2016 | Acute naltrexone does not remediate fronto-striatal disturbances in alcoholic and alcoholic polysubstance-dependent populations during a monetary incentive delay task. | Not conducted in the treatment of AD |
| Nestor et al., 2019 | Naltrexone differentially modulates the neural correlates of motor impulse control in abstinent alcohol-dependent and polysubstance-dependent individuals | Maintenance of abstinence was not the treatment goal |
| Niederhofer et al., 2002 | Acamprosate and its efficacy in treating alcohol dependent adolescents | Retracted article |
| Niederhofer & Staffen, 2003 | Comparison of disulfiram and placebo in treatment of alcohol dependence of adolescents | Included |
| Nimmerrichter et al., 2002 | GHB efficacy in the therapy of alcoholism: clinical evidence. | Maintenance of abstinence was not the treatment goal |
| No author, 2014 | Nalmefene. Alcohol dependence: no advance | Abstract |
| O'Malley et al.,1992 | Naltrexone and Coping Skills Therapy for Alcohol Dependence A Controlled Study | Included |
| O'Malley et al., 1996 | Six-month follow-up of naltrexone and psychotherapy for alcohol dependence | Maintenance of abstinence was not the treatment goal |
| O'Malley et al., 1996 | Naltrexone in the treatment of alcoholism: two-year follow up results. | Not double blind, randomized, placebo-controlled study |
| O’Malley et al., 2003 | Initial and maintenance naltrexone treatment for alcohol dependence using primary care vs specialty care: a nested sequence of 3 randomized trials | Not double blind, randomized, placebo-controlled study |
| O'Malley et al., 2007 | Efficacy of Extended-Release Naltrexone in Alcohol-Dependent Patients Who Are Abstinent Before Treatment | Route of administration different than oral |
| O'Malley et al., 2007 | Naltrexone and Cognitive Behavioral Coping Skills Therapy for the Treatment of Alcohol Drinking and Eating Disorder Features in Alcohol-Dependent Women: A Randomized Controlled Trial | Included |
| O'Malley et al., 2008 | Naltrexone Alone and With Sertraline for the Treatment of Alcohol Dependence in Alaska Natives and Non-Natives Residing in Rural Settings: A Randomized Controlled Trial | Included |
| O'Malley et al., 2009 | Dose-dependent reduction of hazardous alcohol use in a placebo-controlled trial of naltrexone for smoking cessation. | Not conducted in the treatment of AD |
| O'Malley et al., 2015 | Reduction of alcohol drinking in young adults by naltrexone: a double-blind, placebo controlled, randomized clinical trial of efficacy and safety. | Maintenance of abstinence was not the treatment goal |
| O'Malley et al., 2017 | Effect of Varenicline Combined With Medical Management on Alcohol Use Disorder With Comorbid Cigarette Smoking A Randomized Clinical Trial | Maintenance of abstinence was not the treatment goal |
| Oslin et al., 1997 | Tolerability of Naltrexone in Treating Older, Alcohol-Dependent Patients | Abstinence rate not available or available with a different definition Maintenance of abstinence was not the treatment goal |
| Oslin et al., 1997 | Naltrexone as an adjunctive treatment for older patients with alcohol dependence | Abstinence rate not available or available with a different definition |
| Oslin et al., 2002 | Older age predicts better adherence and drinking outcomes. | Not double blind, randomized, placebo-controlled study |
| Oslin et al., 2003 | A functional polymorphism of the mu-opioid receptor gene is associated with naltrexone response in alcohol-dependent patients | Meta-analysis / Reanalysis of already screened studies |
| Oslin, 2005 | Treatment of Late-Life Depression Complicated by Alcohol Dependence | Not double blind, randomized, placebo-controlled study Patients included had psychiatric disorders, poly-addiction or hepatic dysfunctions |
| Oslin et al., 2008 | A Placebo-Controlled Randomized Clinical Trial of Naltrexone in the Context of Different Levels of Psychosocial Intervention | Included |
| Paille et al., 1995 | Double-blind randomized multicentre trial of acamprosate in maintaining abstinence from alcohol | Included |
| Palpacuer et al., 2018 | Pharmacologically controlled drinking in the treatment of alcohol dependence or alcohol use disorders: a systematic review with direct and network meta-analyses on nalmefene, naltrexone, acamprosate, baclofen and topiramate | Meta-analysis / Reanalysis of already screened studies |
| Palpacuer et al., 2019 | Vibration of effects from diverse inclusion/exclusion criteria and analytical choices: 9216 different ways to perform an indirect comparison meta-analysis | Not conducted in the treatment of AD |
| Pani et al., 2014 | Anticonvulsants for alcohol dependence (review) | Meta-analysis / Reanalysis of already screened studies |
| Paparrigopoulos et al., 2010 | An open pilot study of tiagabine in alcohol dependence: tolerability and clinical effects. | Not double blind, randomized, placebo-controlled study |
| Paparrigopoulos et al., 2010 | Treatment of alcohol dependence with low-dose topiramate: an open-label controlled study. | Not double blind, randomized, placebo-controlled study |
| Pelc et al., 1992 | Calcium-acetylhomotaurinate for maintaining abstinence in weaned alcoholic patients: a placebo-controlled double-blind multicenter study | Included |
| Pelc et al., 1997 | Efficacy and safety of acamprosate in the treatment of detoxified alcohol-dependent patients A 90-day placebo-controlled dose-finding study | Included |
| Peng et al, 2018 | Predicting smoking abstinence with biological and self-report measures of adherence to varenicline: Impact on pharmacogenetic trial outcomes | Not conducted in the treatment of AD |
| Perney et al., 2012 | Sleep disturbance in alcoholism: proposal of a simple measurement, and results from a 24-week randomized controlled study of alcohol-dependent patients assessing acamprosate efficacy | Maintenance of abstinence was not the treatment goal |
| Perney et al., 2018 | Insomnia in Alcohol-Dependent Patients: Prevalence, Risk Factors and Acamprosate Effect: An Individual Patient Data Meta-Analysis | Maintenance of abstinence was not the treatment goal |
| Petrakis et al., 2004 | Naltrexone augmentation of neuroleptic treatment in alcohol abusing patients with schizophrenia | Patients included had psychiatric disorders, poly-addiction or hepatic dysfunctions |
| Petrakis et al., 2005 | Naltrexone and disulfiram in patients with alcohol dependence and comorbid psychiatric disorders. | Patients included had psychiatric disorders, poly-addiction or hepatic dysfunctions |
| Petrakis et al., 2006 | Naltrexone and disulfiram in patients with alcohol dependence and comorbid post-traumatic stress disorder. | Patients included had psychiatric disorders, poly-addiction or hepatic dysfunctions |
| Petrakis et al., 2007 | Naltrexone and disulfiram in patients with alcohol dependence and current depression. | Patients included had psychiatric disorders, poly-addiction or hepatic dysfunctions |
| Pettinati et al., 2001 | Double-blind clinical trial of sertraline treatment for alcohol dependence. | Patients included had psychiatric disorders, poly-addiction or hepatic dysfunctions |
| Pettinati et al., 2006 | The Status of Naltrexone in the Treatment of Alcohol Dependence Specific Effects on Heavy Drinking | Meta-analysis / Reanalysis of already screened studies |
| Pettinati et al., 2008 | Gender differences with high-dose naltrexone in patients with co-occurring cocaine and alcohol dependence | Patients included had psychiatric disorders, poly-addiction or hepatic dysfunctions |
| Pettinati et al., 2008 | A double blind, placebo-controlled trial that combines disulfiram and naltrexone for treating co-occurring cocaine and alcohol dependence | Patients included had psychiatric disorders, poly-addiction or hepatic dysfunctions |
| Pettinati et al., 2010 | A Double-Blind, Placebo-Controlled Trial Combining Sertraline and Naltrexone for Treating Co-Occurring Depression and Alcohol Dependence | Patients included had psychiatric disorders, poly-addiction or hepatic dysfunctions |
| Pettinati et al., 2011 | Efficacy of Extended-Release Naltrexone in Patients with Relatively Higher Severity of Alcohol Dependence | Route of administration different than oral |
| Pfeifer et al., 2019 | Efficacy of Varenicline in Patients With Severe Alcohol Dependence: A Pilot Double-Blind Randomized and Controlled Study | Abstinence rate not available or available with a different definition |
| Pierce et al., 2018 | Efficacy, tolerability, and safety of low-dose and high-dose baclofen in the treatment of alcohol dependence: A systematic review and meta-analysis | Meta-analysis / Reanalysis of already screened studies |
| Plebani et al., 2013 | Results from a pilot clinical trial of varenicline for the treatment of alcohol dependence | Maintenance of abstinence was not the treatment goal |
| Plosker, 2015 | Acamprosate: A Review of Its Use in Alcohol Dependence | Meta-analysis / Reanalysis of already screened studies |
| Poldrugo, 1997 | Acamprosate treatment in a long-term community-based alcohol rehabilitation programme | Included |
| Ponizovsky et al., 2015 | Baclofen as add-on to standard psychosocial treatment for alcohol dependence: A randomized, double-blind, placebo-controlled trial with one year follow-up | Maintenance of abstinence was not the treatment goal |
| Powell et al., 1995 | A double-blind, placebo-controlled study of nortriptyline and bromocriptine in male alcoholics subtyped by comorbid psychiatric disorders. | Patients included had psychiatric disorders, poly-addiction or hepatic dysfunctions |
| Preti, 2007 | New developments in the pharmacotherapy of cocaine abuse. | Not conducted in the treatment of AD |
| Rabin et al., 2016 | Does Cannabis Use Moderate Smoking Cessation Outcomes in Treatment-Seeking Tobacco Smokers? Analysis From a Large Multi-Center Trial | Not conducted in the treatment of AD |
| Ralevski et al., 2011 | Treatment With Acamprosate in Patients With Schizophrenia Spectrum Disorders and Comorbid Alcohol Dependence. | Patients included had psychiatric disorders, poly-addiction or hepatic dysfunctions |
| Ralevski et al., 2014 | Quality of life in veterans with alcohol dependence and co-occurring mental illness. | Meta-analysis / Reanalysis of already screened studies |
| Ray et al., 2009 | Naltrexone for the treatment of alcohol dependence among African Americans: results from the COMBINE Study | Meta-analysis / Reanalysis of already screened studies |
| Ray et al., 2011 | A human laboratory study of the effects of quetiapine on subjective intoxication and alcohol craving | Maintenance of abstinence was not the treatment goal |
| Reynaud et al., 2017 | A Randomized, Placebo-Controlled Study of High-Dose Baclofen in Alcohol-Dependent Patients—The ALPADIR Study | Included |
| Richter et al., 2012 | Efficacy and Safety of Levetiracetam for the Prevention of Alcohol Relapse in Recently Detoxified Alcohol-Dependent Patients A Randomized Trial | Included |
| Roberts et al., 1999 | Factor structure and predictive validity of the Obsessive Compulsive Drinking Scale | Maintenance of abstinence was not the treatment goal |
| Roerecke et al., 2015 | Clinical relevance of nalmefene versus placebo in alcohol treatment: Reduction in mortality risk | Meta-analysis / Reanalysis of already screened studies |
| Rohsenow et al., 2000 | Predictors of compliance with naltrexone among alcoholics | Maintenance of abstinence was not the treatment goal |
| Rohsenow et al., 2000 | Naltrexone's effects on reactivity to alcohol cues among alcoholic men | Not conducted in the treatment of AD |
| Rohsenow et al., 2017 | Varenicline versus Nicotine Patch with Brief Advice for Smokers with Substance Use Disorders with or without Depression: Effects on Smoking, Substance Use and Depressive Symptoms | Not conducted in the treatment of AD |
| Rösner et al., 2010 | Acamprosate for alcohol dependence (Review) | Meta-analysis / Reanalysis of already screened studies |
| Rousseaux, 1996 | Does acamprosate diminish the appetite for alcohol in weaned alcoholics? [French article] | Publication not in English |
| Rubio et al., 2001 | Naltrexone versus acamprosate: One year follow-up of alcohol dependence treatment. | Not double blind, randomized, placebo-controlled study |
| Rubio et al., 2002 | Naltrexone improves outcome of a controlled drinking program. | Not double blind, randomized, placebo-controlled study |
| Rubio et al., 2009 | Modulation of impulsivity by topiramate: implications for the treatment of alcohol dependence. | Abstinence rate not available or available with a different definition |
| Ryan et al., 2017 | A Phase 2, Double-Blind, Placebo-Controlled Randomized Trial Assessing the Efficacy of ABT-436, a Novel V1b Receptor Antagonist, for Alcohol Dependence | Maintenance of abstinence was not the treatment goal |
| Saloum et al., 2005 | Efficacy of valproate maintenance in patients with bipolar disorder and alcoholism: a double-blind placebo-controlled study. | Patients included had psychiatric disorders, poly-addiction or hepatic dysfunctions |
| Sass et al., 1996 | Relapse Prevention by Acamprosate Results From a Placebo-Controlled Study on Alcohol Dependence | Included |
| Savulich et al., 2017 | Effects of naltrexone are influenced by childhood adversity during negative emotional processing in addiction recovery | Not conducted in the treatment of AD |
| Schacht et al., 2011 | Neurocognitive performance, alcohol withdrawal, and effects of a combination of flumazenil and gabapentin in alcohol dependence. | Meta-analysis / Reanalysis of already screened studies |
| Schmidt et al., 2007 | Results from two pharmacotherapy trials show alcoholic smokers were more severely alcohol dependent but less prone to relapse than alcoholic non-smokers. | Meta-analysis / Reanalysis of already screened studies |
| Schmitz et al., 2009 | High-dose naltrexone therapy for cocaine-alcohol dependence. | Not conducted in the treatment of AD |
| Schmitz et al., 2014 | A two-phased screening paradigm for evaluating candidate medications for cocaine cessation or relapse prevention: modafinil, levodopa-carbidopa, naltrexone | Not conducted in the treatment of AD |
| Schottenfeld et al., 2014 | Randomized clinical trial of disulfiram for cocaine dependence or abuse during buprenorphine treatment. | Not conducted in the treatment of AD |
| Schuckit et al., 1985 | A one-year follow-up of men alcoholics given disulfiram. | Not double blind, randomized, placebo-controlled study |
| Scott et al., 2005 | Acamprosate A Review of its Use in the Maintenance of Abstinence in Patients with Alcohol Dependence | Meta-analysis / Reanalysis of already screened studies |
| Sellers et al., 1994 | Clinical efficacy of the 5-HT3 antagonist ondansetron in alcohol abuse and dependence. | Maintenance of abstinence was not the treatment goal |
| Shaw et al., 1987 | Tiapride in the Long-term Management of Alcoholics of Anxious or Depressive Temperament | Abstinence rate not available or available with a different definition |
| Shaw et al., 1994 | Tiapride in the Prevention of Relapse in Recently Detoxified Alcoholics | Abstinence rate not available or available with a different definition |
| Shorter et al., 2013 | The Alpha-1 Adrenergic Antagonist Doxazosin for Treatment of Cocaine Dependence: A Pilot Study | Not conducted in the treatment of AD |
| Sinclair et al., 2001 | Evidence about the use of naltrexone and for different ways of using it in the treatment of alcoholism | Meta-analysis / Reanalysis of already screened studies |
| Smolka et al., 2003 | [Advances in the treatment of alcohol dependence: pharmacological relapse prevention] | Meta-analysis / Reanalysis of already screened studies |
| Sofuoglu et al., 2006 | Effects of topiramate in combination with intravenous nicotine in overnight abstinent smokers | Not conducted in the treatment of AD |
| Soyka et al., 1994 | Acamprosate: A new pharmacotherapeutic approach to relapse prevention in alcoholism—Preliminary data. | Meta-analysis / Reanalysis of already screened studies |
| Soyka et al., 2002 | Use of acamprosate and different kinds of psychosocial support in relapse prevention of alcoholism. Results from a non-blind, multicentre study. | Not double blind, randomized, placebo-controlled study |
| Soyka & Chick, 2003 | Use of acamprosate and opioid antagonists in the treatment of alcohol dependence: a European perspective. | Meta-analysis / Reanalysis of already screened studies |
| Spagnolo et al., 2014 | Effects of naltrexone on neural and subjective response to alcohol in treatment-seeking alcohol-dependent patients | Maintenance of abstinence was not the treatment goal |
| Srisurapanont et Jarusuraisun, 2000 | Opioid antagonists for alcohol dependence. | Meta-analysis / Reanalysis of already screened studies |
| Staner et al., 2006 | Effects of acamprosate on sleep during alcohol withdrawal: A double-blind placebo-controlled polysomnographic study in alcohol-dependent subjects | Not conducted in the treatment of AD |
| Stedman et al., 2010 | A Double-Blind, Placebo-Controlled Study With Quetiapine as Adjunct Therapy With Lithium or Divalproex in Bipolar I Patients With Coexisting Alcohol Dependence | Patients included had psychiatric disorders, poly-addiction or hepatic dysfunctions |
| Stein et al., 2013 | Varenicline for Smoking Cessation Among Methadone-Maintained Smokers: A Randomized Clinical Trial | Not conducted in the treatment of AD |
| Stella et al., 2008 | An open randomized study of the treatment of escitalopram alone and combined with gammahydroxybutyric acid and naltrexone in alcoholic patients. | Not double blind, randomized, placebo-controlled study |
| Streeton & Whelan 2001 | Naltrexone, a relapse prevention maintenance treatment of alcohol dependence: a meta-analysis of randomized controlled trials. | Meta-analysis / Reanalysis of already screened studies |
| Sullivan et al., 2013 | Naltrexone treatment for opioid dependence: does its effectiveness depend on testing the blockade? | Not conducted in the treatment of AD |
| Tapp et al., 2015 | Quetiapine for the treatment of cocaine use disorder | Not conducted in the treatment of AD |
| Tempesta, 1994 | The effectiveness and safety of calcium-acethylhomotaurine (acamprosate) on the maintenance of abstinence in weaned alcoholics | Study not found |
| Tempesta et al., 2000 | Acamprosate and relapse prevention in the treatment of alcohol dependence: a placebo-controlled study. | Included |
| Tidey et al., 2008 | Moderators of naltrexone’s effects on drinking, urge, and alcohol effects in nontreatment-seeking heavy drinkers in the natural environment. | Maintenance of abstinence was not the treatment goal |
| Tiihonen et al., 1996 | Citalopram in the treatment of alcoholism. | Maintenance of abstinence was not the treatment goal |
| Tonnensen et al., 1999 | Effect of preoperative abstinence on poor postoperative outcome in alcohol misusers: randomised controlled trial. | Not double blind, randomized, placebo-controlled study |
| Trevisan et al., 2008 | Alcohol detoxification and relapse prevention using valproic acid versus gabapentin in alcohol dependent patients. | Not double blind, randomized, placebo-controlled study |
| Ulrichsen et al., 2010 | Disulfiram in severe alcoholism—an open controlled study. | Not double blind, randomized, placebo-controlled study |
| Umbricht et al., 2014 | Topiramate for cocaine dependence during methadone maintenance treatment: a randomized controlled trial. | Not conducted in the treatment of AD |
| Umhau et al., 2010 | Effect of acamprosate on magnetic resonance spectroscopy measures of central glutamate in detoxified alcohol-dependent individuals: a randomized controlled experimental medicine study. | Maintenance of abstinence was not the treatment goal |
| Umhau et al., 2011 | Pharmacologically induced alcohol craving in treatment seeking alcoholics correlates with alcoholism severity, but is insensitive to acamprosate | Maintenance of abstinence was not the treatment goal |
| Verheul et al., 2005 | Predictors of acamprosate efficacy: results from a pooled analysis of seven European trials including 1485 alcohol-dependent patients | Meta-analysis / Reanalysis of already screened studies |
| Verplaetse et al., 2019 | Alcohol Abstainer Status and Prazosin Treatment in Association with Changes in Posttraumatic Stress Disorder Symptoms in Veterans with Comorbid Alcohol Use Disorder and Posttraumatic Stress Disorder | Patients included had psychiatric disorders, poly-addiction or hepatic dysfunctions |
| Volpicelli et al., 1992 | Naltrexone in the Treatment of Alcohol Dependence | Abstinence rate not available or available with a different definition |
| Volpicelli et al., 1997 | Naltrexone and Alcohol Dependence Role of Subject Compliance | Included |
| Weiss et al., 2008 | Do patients with alcohol dependence respond to placebo? Results from the COMBINE Study | Meta-analysis / Reanalysis of already screened studies |
| West et al., 2018 | Factors associated with the efficacy of smoking cessation treatments and predictors of smoking abstinence in EAGLES | Not conducted in the treatment of AD |
| Wetzel et al., 2004 | Combination treatment with nefazodone and cognitive-behavioral therapy for relapse prevention in alcohol-dependent men: A randomized controlled study. | Abstinence rate not available or available with a different definition |
| Whitworth et al., 1996 | Comparison of acamprosate and placebo in long-term treatment of alcohol dependence | Included |
| Whyte et al., 1974 | Disulfiram implant: a controlled trial. | Route of administration different than oral |
| Wiesbeck et al., 2001 | Flupenthixol decanoate and relapse prevention in alcoholics: results from a placebo-controlled study | Route of administration different than oral |
| Wilde & Wagstaff, 1997 | Acamprosate A Review of its Pharmacology and Clinical Potential in the Management of Alcohol Dependence After Detoxification | Meta-analysis / Reanalysis of already screened studies |
| Wilson et al., 1976 | Disulfiram implantation: placebo, psychological deterrent, and pharmacological deterrent effects. | Route of administration different than oral |
| Wilson et al., 1980 | Disulfiram implantation: A trial using placebo implants and two types of controls. | Route of administration different than oral |
| Winstock et al., 2009 | Lithium carbonate in the management of cannabis withdrawal in humans: an open-label study | Not conducted in the treatment of AD |
| Witkiewitz et al., 2012 | Acamprosate for treatment of alcohol dependence: mechanisms, efficacy, and clinical utility | Meta-analysis / Reanalysis of already screened studies |
| Wölver et al., 2011 | The effects of combined acamprosate and integrative behaviour therapy in the outpatient treatment of alcohol dependence: a randomized controlled trial | Abstinence rate not available or available with a different definition Not double-blind, randomized, placebo-controlled study |
| Worley et al., 2018 | Predictors of reduced smoking quantity among recovering alcohol dependent men in a smoking cessation trial | Maintenance of abstinence was not the treatment goal |
| Yoshimura et al., 2014 | Efficacy of disulfiram for the treatment of alcohol dependence assessed with a multicenter randomized controlled trial. | Not double blind, randomized, placebo-controlled study |
| Zarkin et al., 2008 | Cost and cost-effectiveness of the COMBINE study in alcohol-dependent patients | Meta-analysis / Reanalysis of already screened studies |
| Zawertailo et al., 2020 | Safety and Efficacy of Varenicline for Smoking Cessation in Alcohol-Dependent Smokers in Concurrent Treatment for Alcohol Use Disorder: A Pilot, Randomized Placebo-Controlled Trial | Maintenance of abstinence was not the treatment goal |
| Unknown author, 2013 | Baclofen and severe alcohol dependence: an uncertain harm-benefit balance as of early 2013 | Meta-analysis / Reanalysis of already screened studies |
| Unknown author, 2014 | Nalmefene. Alcohol dependence: no advance | Meta-analysis / Reanalysis of already screened studies |

## Supplementary information: Assessment of risk of bias in included studies

The risk of bias assessment for each RCT in this review was performed using the criteria recommended by the Cochrane Handbook (Higgins et al., 2020). This is achieved by answering a prespecified question about the adequacy of the study in relation to the entry, such that a judgement of “Yes” (“+” in the summary table) indicates low risk of bias, “No” (“-” in the summary table) indicates high risk of bias, and “Unclear” (“?” in the summary table) indicates unclear or unknown risk of bias. To make these judgments we used the criteria indicated by the handbook adapted to the addiction field. In particular, the following criteria were used to evaluate risk of bias:

1. Sequence generation (avoidance of selection bias);

2. Allocation concealment (avoidance of selection bias);

3. Completeness of outcome data (avoidance of attrition bias);

4. Selective reporting (avoidance of reporting bias);

5. Other possible bias, such as similarity of patients in the groups (avoidance of selection bias);

6. Blinding of patients, providers and of subjective outcomes (avoidance of performance bias and detection bias).

Inclusion of all patients in the analysis and drop out rate were used to evaluate completeness of outcome data. Mentioning the study protocol in the paper was used to evaluate the possibility of selective reporting. Similarity of patients in the groups at the starting of the trial and methods used to increase the strength of the statistical analysis were used to check other possible bias. Blinding was considered separately for patients, providers and assessors of subjective outcomes.

Results of risk of bias evaluation are illustrated in the Risk of bias table (Table S6).

**Table S6 Methodological quality summary: review authors’ judgements about methodological quality items for each included study.**
